# Loss of the *Caenorhabditis elegans* pocket protein LIN-35 reveals MuvB's innate function as the repressor of DREAM target genes

**DOI:** 10.1371/journal.pgen.1007088

**Published:** 2017-11-01

**Authors:** Paul D. Goetsch, Jacob M. Garrigues, Susan Strome

**Affiliations:** Department of Molecular, Cell and Developmental Biology, University of California Santa Cruz, Santa Cruz, California, United States of America; University of Wuerzburg, GERMANY

## Abstract

The DREAM (Dp/Retinoblastoma(Rb)-like/E2F/MuvB) transcriptional repressor complex acts as a gatekeeper of the mammalian cell cycle by establishing and maintaining cellular quiescence. How DREAM’s three functional components, the E2F-DP heterodimer, the Rb-like pocket protein, and the MuvB subcomplex, form and function at target gene promoters remains unknown. The current model invokes that the pocket protein links E2F-DP and MuvB and is essential for gene repression. We tested this model by assessing how the conserved yet less redundant DREAM system in *Caenorhabditis elegans* is affected by absence of the sole *C*. *elegans* pocket protein LIN-35. Using a LIN-35 protein null mutant, we analyzed the assembly of E2F-DP and MuvB at promoters that are bound by DREAM and the level of expression of those "DREAM target genes" in embryos. We report that LIN-35 indeed mediates the association of E2F-DP and MuvB, a function that stabilizes DREAM subunit occupancy at target genes. In the absence of LIN-35, the occupancy of E2F-DP and MuvB at most DREAM target genes decreases dramatically and many of those genes become upregulated. The retention of E2F-DP and MuvB at some target gene promoters in *lin-35* null embryos allowed us to test their contribution to DREAM target gene repression. Depletion of MuvB, but not E2F-DP, in the sensitized *lin-35* null background caused further upregulation of DREAM target genes. We conclude that the pocket protein functions primarily to support MuvB-mediated repression of DREAM targets and that transcriptional repression is the innate function of the evolutionarily conserved MuvB complex. Our findings provide important insights into how mammalian DREAM assembly and disassembly may regulate gene expression and the cell cycle.

## Author summary

The 8-subunit DREAM transcriptional repressor complex contains 3 functional components that together control expression of cell cycle and developmental genes. How the E2F-DP transcription factor heterodimer, the pocket protein, and the highly conserved MuvB complex coalesce on chromatin and repress DREAM target genes has yet to be determined. We directly tested the prevailing model that the DREAM pocket protein links E2F-DP to MuvB and is required for gene repression. Using a protein null mutant of the sole *C*. *elegans* pocket protein LIN-35, we demonstrate that the pocket protein indeed links E2F-DP and MuvB, which aids in the stable occupancy of DREAM components near target genes. Depletion of additional DREAM components in *lin-35* null worms revealed that the remaining chromatin-bound MuvB represses target genes. We conclude that the MuvB subcomplex mediates DREAM’s critical repressive function. Our functional genomics approach in the simplified *C*. *elegans* system reveals that the ancestral function of the pocket protein is to stabilize the innate repressive activity of MuvB, ensuring proper regulation of DREAM target genes through development.

## Introduction

As embryonic cells develop and differentiate, a conserved transcriptional program establishes their ordered exit from the cell cycle into a resting phase, also called G_0_ or quiescence. The highly conserved DREAM (for Dp, Retinoblastoma (Rb)-like, E2F, and MuvB) transcriptional repressor complex mediates this cell cycle quiescence program [1–3]. DREAM contains 3 components: a “repressive” E2F-DP heterodimer (in mammals, E2F4/5-DP1/2), a retinoblastoma-like pocket protein (in mammals, p130 or p107), and a 5-subunit subcomplex called MuvB (in mammals, LIN9, LIN37, LIN52, LIN54, and RBAP48) [1, 4, 5]. Together, the 8-subunit DREAM complex negatively regulates cell cycle reentry by directly repressing key cell cycle genes [1, 2]. How DREAM assembly culminates in gene repression is not understood.

The prevailing model for DREAM complex activity is that DREAM assembly at promoters, driven by p130/p107 linkage of E2F-DP and MuvB, mediates gene repression. Biochemical analyses in mammalian cells have revealed that DREAM assembly is triggered by DYRK1A phosphorylation of the MuvB subunit LIN52, directing MuvB association with the pocket protein [6, 7]. The p130/p107 pocket domain simultaneously interacts with MuvB and the transactivation domain of repressive E2F-DP through separate binding interfaces, completing assembly of the complex [8]. To repress transcription, DREAM localizes to chromatin through E2F-DP and the MuvB subunit LIN54 binding to DNA sequence motifs called cell cycle-dependent elements (CDEs) and cell cycle genes homology regions (CHRs), respectively [9–13]. Loss-of-function analyses in mice support a central role for the pocket protein in DREAM complex activity, since both p130/p107 double knockout mice and E2F4/5 double knockout mice display neonatal lethality, which has been attributed to defects in chondrocyte proliferation [14–16]. However, no direct evidence has demonstrated how E2F-DP, the pocket protein, and MuvB coordinate to repress target genes.

Because of the transcriptional dynamics observed during the cell cycle, how each component, especially MuvB, contributes to gene repression remains obscure. Upon progression from G_0_ into the cell cycle, p130 is phosphorylated by CDK4/6-cyclin D and dissociates from DREAM [2, 7, 17]. The transcription factor B-Myb then binds MuvB, forming the Myb-MuvB (MMB) complex, which activates late cell cycle genes [18–22]. This dual role for MuvB in repressing and activating genes, depending on the cell cycle context, complicates studies that have attempted to address its role in DREAM gene repression. For example, in contrast to E2F-DP and pocket protein loss-of-function, loss of the MuvB subunit LIN9 in mice causes early embryonic lethality [23]. This phenotype is likely due to MuvB’s activating role in MMB in the late cell cycle and not its role in DREAM [23]. Therefore, how MuvB’s inclusion in the DREAM complex contributes to target gene repression remains an outstanding question [2].

**Fig 1:**
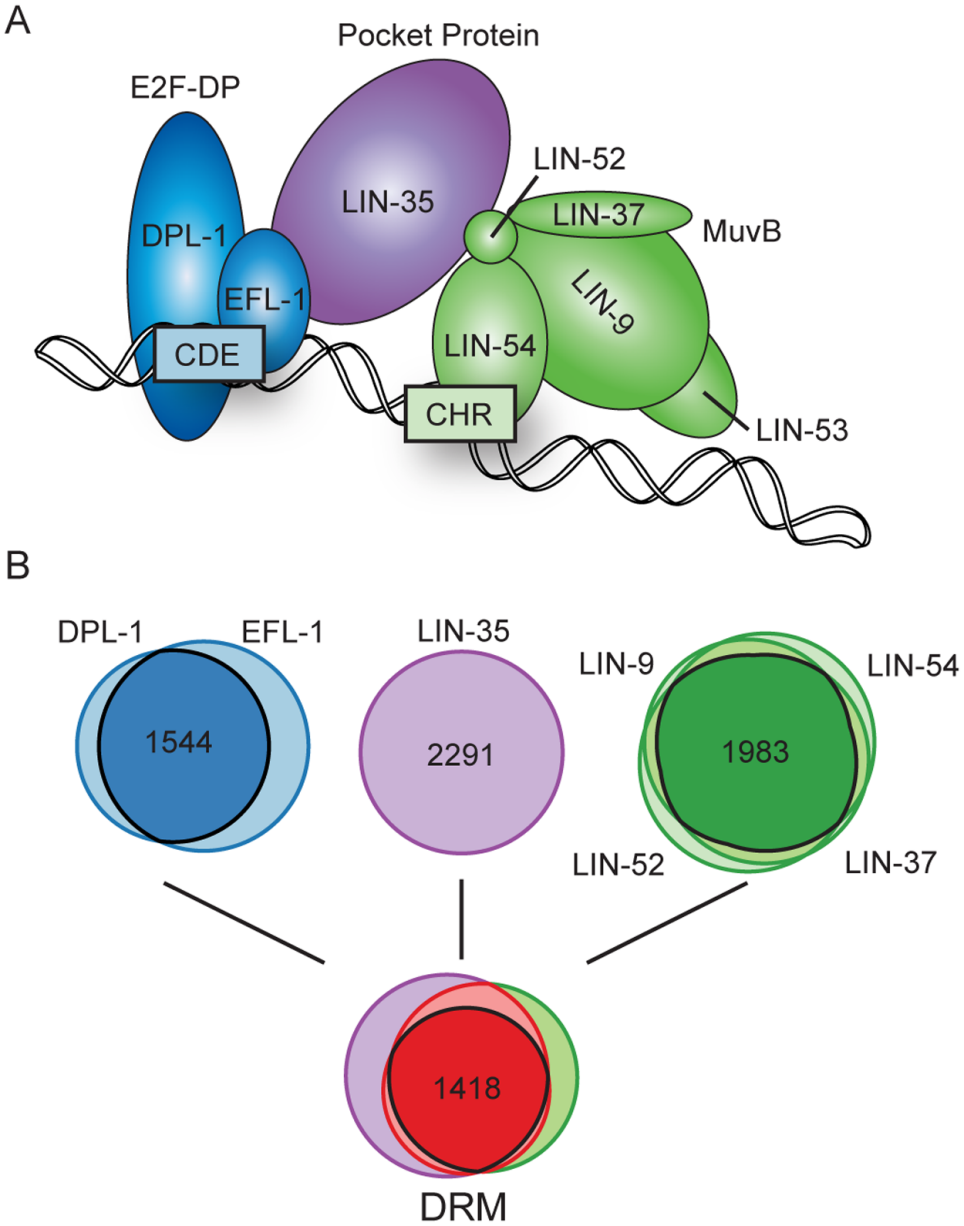
Chromatin association of the DRM complex in late *C*. *elegans* embryos. (A) Model of the *C*. *elegans* DRM complex bound to DNA: E2F-DP (blue), the pocket protein LIN-35 (purple), and the 5-subunit MuvB core (green). Subunit interactions and binding of E2F-DP and LIN-54 to CDE and CHR DNA motifs, respectively, are based on [7, 11]. (B) Tier 1: Venn diagrams showing ChIP-seq peak overlaps in wild-type late embryos for E2F-DP (1771 DPL-1 peaks, 2119 EFL-1 peaks), LIN-35 (2291 peaks), and 4 of 5 subunits of the MuvB core (2067 LIN-9 peaks, 2111 LIN-37 peaks, 2284 LIN-52 peaks, 2290 LIN-54 peaks). Tier 2: Venn diagram showing the total peak overlaps between E2F-DP, LIN-35, and MuvB.

In *C*. *elegans*, the homologous complex, called DRM, acts similarly to the mammalian DREAM complex on chromatin (Fig 1A). *C*. *elegans* possesses only one pocket protein, LIN-35, which functionally resembles p130/p107 [24]. *C*. *elegans* E2F-DP (EFL-1-DPL-1) resembles the repressive E2F4-DP1 heterodimer [25]. Importantly, *C*. *elegans* MuvB likely acts predominantly or solely in a repressive capacity, because *C*. *elegans* LIN-52 lacks the phosphorylation switch present in mammalian LIN52 and no *C*. *elegans* B-Myb homolog has been identified [7, 26]. A key role of the *C*. *elegans* DRM complex is to keep germline genes repressed in developing somatic cells, as mutations in 7 of the 8 subunits cause ectopic expression of germline genes in somatic cells [27, 28]. Thus, loss-of-function analyses of *C*. *elegans* DRM subunits are well suited to address how E2F-DP, the pocket protein, and MuvB coordinate on chromatin to repress DRM target genes.

Here we utilize a protein null mutant of the sole *C*. *elegans* pocket protein LIN-35 to test how each DRM component contributes to target gene repression. Using co-immunoprecipitation, we demonstrate that LIN-35 mediates E2F-DP and MuvB association. Using chromatin immunoprecipitation linked to high throughput sequencing (ChIP-seq), we demonstrate that E2F-DP and MuvB continue to localize to chromatin in *lin-35* null embryos, but their occupancy is reduced globally. Thus, DRM components can assemble on chromatin in the absence of LIN-35 bridging E2F-DP and MuvB association, but LIN-35 is required for maximal DRM chromatin occupancy on all regulatory elements. We also determine that misregulation of many genes with promoter-bound DRM, or “DRM target genes,” begins in *lin-35* null late-stage embryos. DRM target genes that retain some DRM chromatin occupancy in the absence of LIN-35 remain at least partially repressed; repression is mediated by MuvB, as revealed by upregulation of those target genes when MuvB components are depleted from *lin-35* null mutants. Loss of MuvB activity in *lin-35* null worms also results in sterility. Our findings highlight that MuvB's innate function is as a transcriptional repressor of DRM target genes and provide evidence for how this function is facilitated by LIN-35. These results shed light on how DRM components coordinate gene repression in worms, which has important implications for the current model of DREAM-mediated gene repression in mammals.

## Results

### E2F-DP and MuvB act primarily within the repressive DRM complex in *C*. *elegans* embryos

Chromatin association of the 8-subunit DRM complex occurs through the sequence-specific DNA-binding activities of E2F-DP and LIN-54 (Fig 1A) [11]. To begin to address how E2F-DP, LIN-35, and MuvB contribute to transcriptional repression, we re-evaluated each DRM subunit’s *in vivo* chromatin-bound landscape in *C*. *elegans* late embryos. Expanding upon our previously published 2 replicates of ChIP-seq data [29], we performed an additional biological replicate of each E2F-DP and MuvB subunit in wild-type (WT) late embryo extracts. Late embryos represent a predominantly somatic cell sample, as only 2 germ cells (Z2/Z3) are present in each 200- to 550-cell late embryo. LIN-53 was not included, because its chromatin localization was not as robust as the other MuvB subunits [29]. We applied a 1% Irreproducible Discovery Rate (IDR) threshold on peaks called from the 3 biological replicates to identify the high-confidence chromatin binding sites for each individual DRM subunit (S1 Table). The overlap of individual subunit peaks defined 1544 E2F-DP peaks (EFL-1 and DPL-1 overlap) and 1983 MuvB peaks (at least 3 of 4 subunit overlap). The overlap between E2F-DP, MuvB, and 2291 LIN-35 peaks defined 1418 high-confidence DRM binding sites that were used for all subsequent analyses (Fig 1B). We did not observe any appreciable chromatin association of individual subunits or sub-complexes independent of DRM in late embryos (S1 Fig). To determine whether DRM subunits preferentially assemble with other transcription factors on high occupancy target (HOT) regions, we compared our DRM peak regions with HOT regions observed in *C*. *elegans* embryos [30]. We observed that 20–30% of individual subunit peaks and 425 of the 1418 high-confidence DRM binding regions coincide with embryonic HOT regions (S1 Table). Overall, we conclude that E2F-DP and MuvB operate primarily within the context of the repressive DRM complex during late *C*. *elegans* embryogenesis, which represents an ideal stage for evaluating the mechanism of DRM action.

### The *C*. *elegans* pocket protein LIN-35 mediates the association of E2F-DP and MuvB

**Fig 2:**
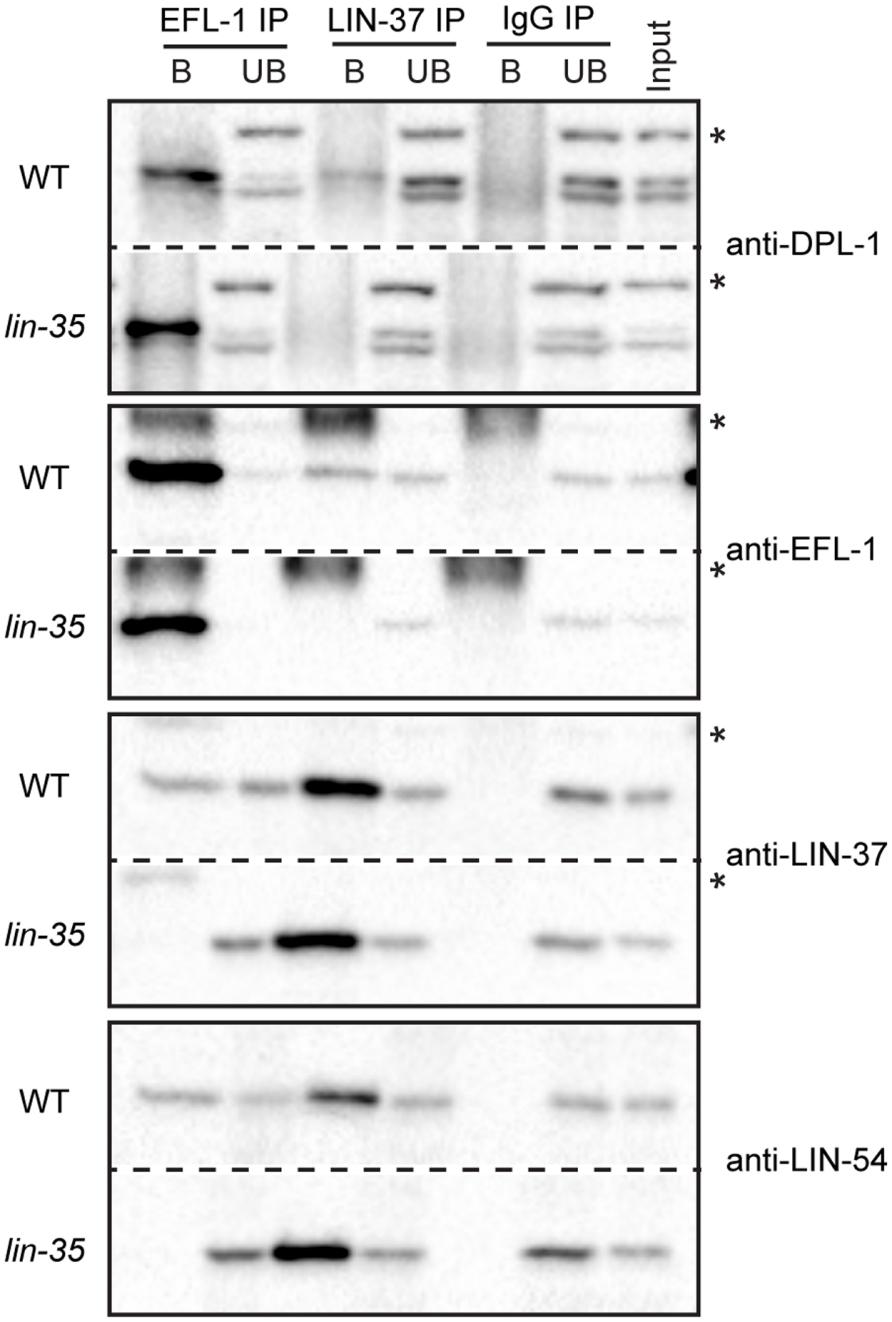
Co-immunoprecipitation analysis of DRM components from wild-type and *lin-35* null late embryo cell lysates. Late embryo extracts from wild-type (WT) and a *lin-35* null mutant were immunoprecipitated with anti-EFL-1, anti-LIN-37, or control IgG antibodies. Proteins bound (B) and unbound (UB) were separated by SDS/PAGE, and western blot analysis was performed using the antibodies indicated on the right. 5% of Input was included. Asterisks indicate non-specific bands. Full blots are shown in S2 Fig.

According to the current model of DRM complex assembly (Fig 1A), LIN-35 acts as a scaffold through concurrent interactions with EFL-1 and LIN-52 on opposite faces of the pocket domain [7, 16]. To address how LIN-35 contributes to DRM complex formation in *C*. *elegans*, we performed co-immunoprecipitation (co-IP) experiments from WT and *lin-35(n745)* late embryo protein extracts (Fig 2). The *n745* allele introduces an early stop codon and is a protein-null allele [24]. EFL-1 immunoprecipitation successfully pulled down the MuvB components LIN-37 and LIN-54 from WT extracts but not from *lin-35* null extracts. In reciprocal pull-downs, LIN-37 immunoprecipitation successfully pulled down EFL-1 and DPL-1 from WT extracts but not from *lin-35* null extracts. As controls, the EFL-1 and LIN-37 IPs successfully pulled down their respective complex partners DPL-1 or LIN-54 from both extracts. These results were observed in 2 biological replicate experiments (S2 Fig) and demonstrate that E2F-DP and MuvB association requires LIN-35.

### Loss of LIN-35 impairs but does not eliminate E2F-DP and MuvB chromatin association

**Fig 3:**
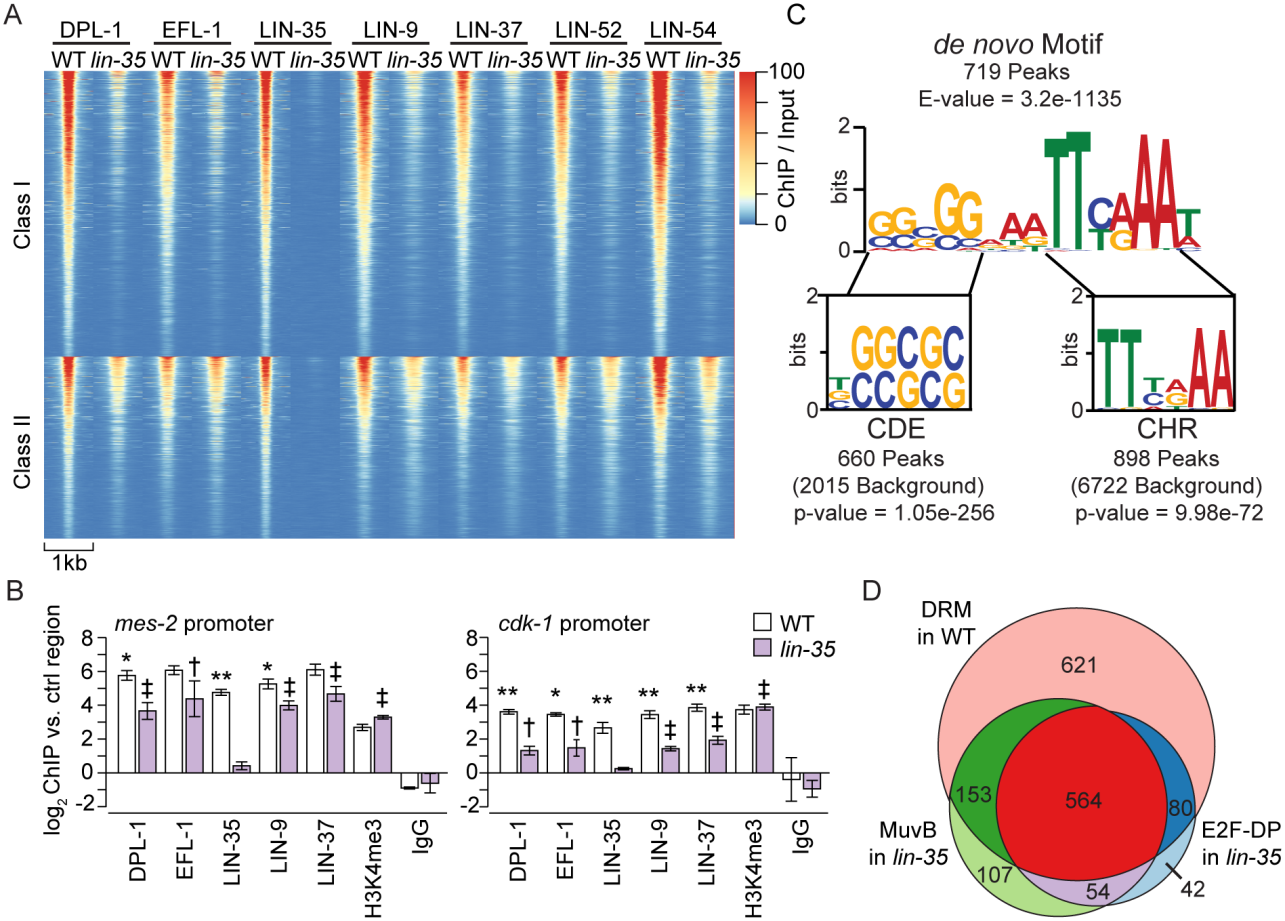
Comparison of DRM subunit chromatin localization in wild-type and *lin-35* null late embryos. (A) Heatmap of normalized ChIP-seq profiles of pooled replicates for each DRM subunit sorted from highest to lowest signal in the following classes. Class I: DRM peak occupancy significantly decreased in *lin-35* compared to wild type (WT) for at least 1 subunit (866 peaks). Class II: DRM peak occupancy not significantly decreased in *lin-35* compared to WT (552 peaks). Significance was determined using DEseq2 with an FDR < 0.05. (B) ChIP-qPCR of 5 DRM subunits at 2 Class II peaks in the *mes-2* and *cdk-1* promoters. H3K4me3 ChIP was included as a positive control. Signals are presented as the log_2_ fold enrichment of the ChIP signal for each region vs. a negative control region (a non-coding region of chromosome IV). Error bars indicate standard error of the mean. Significance was determined by a student’s T test between subunit ChIP values in WT versus *lin-35* (* p-value < 0.05, ** p-value < 0.01) or between subunit versus IgG ChIP values in *lin-35* († p-value < 0.05, ‡ p-value < 0.01). (C) Sequence logo of the DRM motif observed *de novo* in 719 DRM peaks using MEME. Aligned below are the sequence logos of the conserved CDE and CHR motifs observed in 660 DRM peaks and 898 DRM peaks, respectively. The p-value for the conserved motif enrichment over background was calculated using a hypergeometric distribution. Additional motif enrichment analyses are shown in S6 Fig. (D) Venn diagram showing ChIP-seq peak overlaps in late embryos for DRM in WT (pink, 1418 peaks), MuvB in *lin-35* (green, 878 peaks), and E2F-DP in *lin-35* (blue, 740 peaks).

To test if loss of LIN-35 affects E2F-DP and/or MuvB chromatin localization, we performed ChIP-seq of E2F-DP (DPL-1 and EFL-1) and MuvB (LIN-9, LIN-37, LIN-52, and LIN-54) on 3 biological replicates of *lin-35* null late embryos. LIN-35 ChIP-seq was performed on 2 *lin-35* null biological replicates as a negative control. We observed a genome-wide decrease in E2F-DP and MuvB subunit chromatin occupancy in *lin-35* null embryo extracts (Fig 3A, S3A Fig). To define the reproducible differences between *lin-35* null and WT DRM subunit ChIP-seq, we used DEseq2 differential binding analysis [31]. Of the 1418 high-confidence DRM peaks identified in WT (Fig 1B, S1 Table), 866 peaks displayed significantly decreased chromatin occupancy by at least 1 E2F-DP/MuvB subunit in *lin-35* null (Class I, FDR < 0.05) (Fig 3A). 71% of Class I peaks showed a significant decrease in chromatin occupancy of at least 3 of the 6 analyzed subunits in *lin-35* null compared to WT, and 27% showed a significant decrease for all 6 subunits. Of the remaining 552 peaks that were not significantly decreased (Class II), 6 showed a significant increase in chromatin occupancy of at least one subunit; this corresponds to 0.4% of all high-confidence DRM peaks. Our data demonstrate that in the absence of LIN-35, the chromatin association of E2F-DP and MuvB is significantly reduced (Fig 3A, S3A Fig).

Although the 552 Class II peaks did not meet our significance cut-off for being reduced in *lin-35* null compared to WT, these peaks appeared reduced in *lin-35* (Fig 3A). In fact, the signal from 2 of the 3 *lin-35* null ChIP-seq replicates suggested that LIN-35 loss greatly affected DRM subunit occupancy at all sites (S4 Fig). When we removed the remaining *lin-35* null ChIP-seq replicate from the differential binding analysis, then 93% of all DRM peaks displayed significantly decreased chromatin occupancy by at least one DRM subunit, and 64% showed a significant decrease for all 6 subunits, in *lin-35* null compared to WT. Taken together, our genome-wide analysis revealed that E2F-DP and MuvB chromatin occupancy is impaired globally in *lin-35* null embryos.

We validated our ChIP-seq findings by ChIP-qPCR analysis of 8 Class I DRM peaks and 6 Class II peaks. In *lin-35* null embryos, we observed a significant decrease in chromatin occupancy of the majority of DRM subunits at all 14 sites tested (Fig 3B, S3B Fig). Two representative Class II peaks within the *mes-2* and *cdk-1* promoters are shown in Fig 3B. Histone H3 lysine 4 trimethylation (H3K4me3) ChIP was used as a positive control; it confirmed that the observed reduction of DRM subunit chromatin occupancy in *lin-35* null compared to WT late embryos was specific to DRM subunits (Fig 3B, S3B Fig). Similar to our ChIP-seq analysis, in *lin-35* null embryos, the chromatin occupancy of the DRM subunits DPL-1, EFL-1, LIN-9, and LIN-37 was significantly enriched over the IgG negative control near 11 of the 14 promoter regions tested, including *mes-2* and *cdk-1* (Fig 3B, S3B Fig). Interestingly, the DRM complex occupies the promoter region of most of the genes that encode DRM subunits (S5A Fig), suggesting that the DRM complex represses its own expression. Indeed, the majority of DRM subunit transcripts were elevated in the 2 DRM mutants we tested (S5B Fig); protein levels were similar or slightly elevated in *lin-35* null compared to WT late embryos, based on western blot analysis (S5C Fig). These results indicate that the observed decrease in DRM subunit chromatin occupancy was not driven by reduced protein levels. To extend this analysis beyond embryos, we also performed ChIP-qPCR to test DPL-1 and LIN-37 occupancy in L1 larvae. As in embryos, we observed a decrease in DPL-1 and LIN-37 occupancy in *lin-35* null L1 larvae compared to WT L1 larvae (S5D Fig). Together, our ChIP analyses of DRM subunits in the *lin-35* null indicate that E2F-DP and MuvB chromatin occupancy is significantly impaired but not eliminated at most genomic sites in the absence of the pocket protein.

We tested whether the DRM DNA binding motif is enriched in Class I targets and/or Class II targets. We performed *de novo* DNA motif discovery using MEME [32] on the 1418 high-confidence DRM peak regions and identified the characteristic hybrid CDE/CHR motif similar to what has been observed previously (Fig 3C) [33]. Additionally, we performed a search for the known E2F-DP binding motif (CDE, IUPAC code: BSSSSS) and LIN-54 binding motif (CHR, IUPAC code: TTYRAA), restricting our search to phylogenetically conserved motifs similar to an analysis performed on the mammalian genome (S6 Fig) [34]. The analyses together identified 719 peaks with the *de novo* CDE/CHR motif, 660 peaks with a conserved CDE motif, and 898 peaks with a conserved CHR motif (Fig 3C, S1 Table). However, the motif content of a DRM peak does not predict whether E2F-DP or MuvB remain more or less stably bound in the absence of LIN-35 (S6 Fig).

### E2F-DP and MuvB remain confined to WT peak regions in *lin-35* null embryos

In mammalian cells, phosphorylation of the pocket protein triggers DREAM disassembly and reentry into the cell cycle [2, 7]. Activating E2F-DPs replace repressive E2F-DP, and MuvB forms the activating MMB complex to drive cell cycle gene expression [22, 35]. Since *C*. *elegans* E2F-DP alone can activate genes in the germline [36], we assessed whether the chromatin-bound landscape of the DRM components changes in *lin-35* null embryos. We performed peak calling analysis on the *lin-35* null ChIP-seq replicates, as described previously (S2 Table). For each subunit, roughly half of the WT peaks were lost in *lin-35* null embryos (S7 Fig). After overlapping WT high-confidence DRM peaks with E2F-DP and MuvB peaks detected in *lin-35* null embryos, we observed that 621 of the 1418 high-confidence DRM peaks in WT lose detectable E2F-DP and MuvB occupancy in *lin-35* null embryos (Fig 3D). 564 of the 1418 high-confidence DRM peaks in WT retain appreciable E2F-DP and MuvB occupancy in *lin-35* null embryos, underscoring that loss of LIN-35 impairs but does not eliminate DRM complex chromatin localization (Fig 3A). We did not observe any appreciable or consistent appearance of E2F-DP or MuvB at new sites (S7 Fig). Thus, loss of LIN-35 does not appear to result in *de novo* binding of E2F-DP or MuvB to new sites but rather destabilizes binding to their native sites, resulting in elimination of DRM occupancy from about half of the native sites.

### DRM subunit destabilization affects target gene expression in late stage embryos

**Fig 4:**
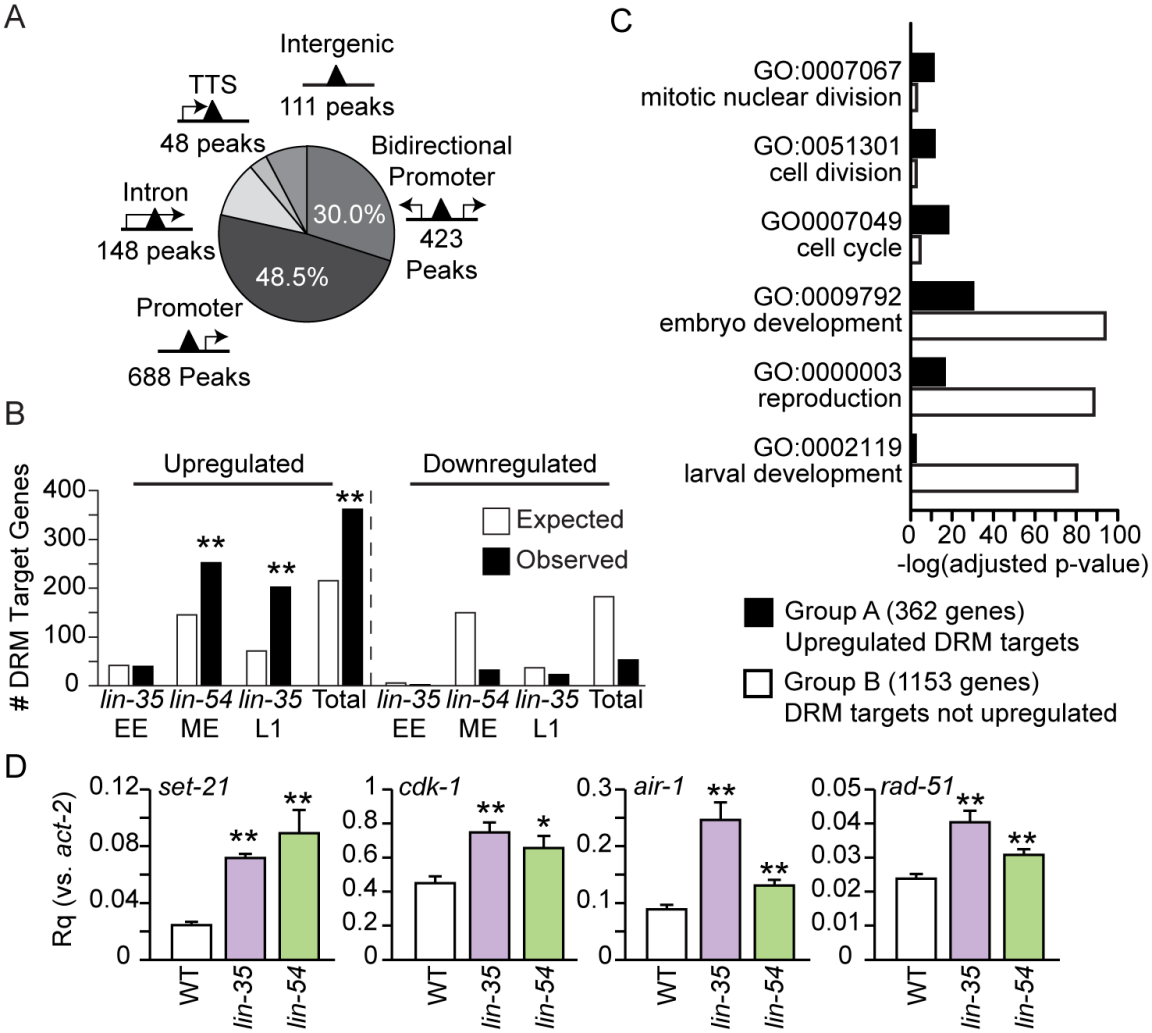
Locations of DRM peaks and misregulation of DRM target genes. (A) Pie chart showing the number of peaks within a promoter (-1000 bp to +100 bp) of bidirectional gene pairs (423 peaks) or single genes (688 peaks); within an intron (148 peaks); at the transcriptional termination site (TTS, 48 peaks); or intergenic (> 1000 bp from a gene, 111 peaks). The percentage of total peaks is indicated for promoter peaks only. (B) Enrichment analysis of DRM target genes expected and observed among genes up- or downregulated in microarray expression analyses reported in [37] (*lin-35* early embryos (EE) and L1s) and [33] (*lin-54* mixed-stage embryos (ME)) and a combination of all 3 experiments (Total). The significance of enrichment observed over expected was determined using a hypergeometric distribution (** p-value < 1x10^-5^). (C) Bar chart plotting the –log_10_ Benjamini adjusted p-value of selected GO term enrichment observed for 362 upregulated DRM target genes (Group A, black) compared to GO term enrichment for 1153 DRM target genes not upregulated (Group B, white). (D) RT-qPCR analysis comparing transcript levels of 4 Group A genes (*set-21*, *cdk-1*, *air-1*, and *rad-51*) in *lin-35(n745)* (purple) and *lin-54(n2231)* (green) late embryos to the level in wild-type (WT, white) late embryos. Expression values from 2 independent experiments each consisting of 4 biological replicates were averaged and are presented as the relative quantity (Rq) compared to *act-2*. Error bars indicate standard error of the mean, and significance was determined by a student’s T test between transcript levels in mutant vs WT (* p-value < 0.05, ** p-value < 0.01). Additional genes are shown in S5 Table.

To address the effects of LIN-35 loss on target gene transcription, we mapped the 1418 high-confidence DRM binding sites to likely gene targets and then analyzed transcript levels from those genes in *lin-35* null mutants. 78% of all DRM binding sites map to within at least one gene’s promoter region, which we defined as 1000 base pairs upstream to 100 base pairs downstream (-1000 bp, +100 bp) of a transcriptional start site (Fig 4A, S3 Table). Of these promoter peaks, 688 DRM binding sites were observed within 677 single gene promoters, and 423 DRM binding sites were observed within bidirectional promoters for 844 genes. Of the remaining DRM binding sites, 148 were observed within an intron of 137 genes, 48 were observed within 1000 bp of 46 transcriptional termination sites, and 111 were observed in intergenic regions (Fig 4A, S3 Table).

To generate a list of high-confidence DRM targets, we assessed whether genes with DRM bound in their promoter region in WT (DRM target genes) were misregulated in previously published expression analyses that compared *lin-35* null to WT in early embryos and L1 larvae [37] and compared *lin-54(n2990)* mutant to WT in mixed-stage embryos [33] (S3 Table) using microarray analysis. The *lin-54* allele produces protein with impaired DNA-binding ability, which reduces its chromatin occupancy by ~50% [33]. Upregulated genes in both *lin-35* null L1s and *lin-54* mutant mixed embryos were significantly enriched for DRM target genes (Fig 4B). Our analysis defined 362 "high-confidence DRM targets" (bound by DRM and upregulated in DRM mutants), which we refer to as Group A DRM targets (Fig 4B, S3 Table, S8A Fig). The remaining "lower-confidence DRM targets" (1153 genes) we refer to as Group B DRM targets, because we do not have evidence that DRM represses these genes.

To investigate why the expression of many DRM target genes appears to be unaffected by disruption of DRM activity, we performed a Gene Ontology (GO) enrichment analysis of the 362 Group A genes and the 1153 Group B genes using DAVID (Fig 4C, S4 Table) [38, 39]. REViGO was applied to remove redundant GO terms (S9A Fig) [40]. Group A includes genes for cell cycle, while Group B lacks those genes; both groups include genes for development and reproduction (Fig 4C). DRM target genes marked by Class I peaks, Class II peaks, or HOT regions were not enriched for any particular GO term (S9 Fig). We speculate that the genes in Group A and Group B have distinct requirements for transcriptional activation: Group A genes, which include cell cycle genes that are likely expressed in all cell types, turn on when DRM activity is lost, while Group B genes do not turn on when DRM activity is lost, perhaps because they require additional transcriptional activators that are present only at specific developmental stages and/or in specific cell types.

Since developmental stage appears to greatly affect whether loss of DRM function causes target gene misregulation (Fig 4B, S8B Fig), we performed RT-qPCR analysis on *lin-35* null and *lin-54(n2231)* mutant late embryos to match our ChIP-seq analysis. The *lin-54(n2231)* allele contains the same point mutation as *lin-54(n2990)*, which impairs its DNA binding [33]. We included *lin-54* late embryos to address whether the difference in number of genes significantly upregulated in *lin-35* null early embryos and *lin-54* mutant mixed-stage embryos (Fig 4B) is due to their different stages. We tested 4 candidates from Group A DRM target genes, which we expected to be upregulated in late embryos: *set-21*, *cdk-1*, *air-1* and *rad-51*. All 4 candidates were significantly upregulated in both *lin-35* null and *lin-54* mutant late embryos as compared to WT late embryos (Fig 4D). Out of a total of 25 Group A genes tested, 22 were significantly upregulated in *lin-35* null and/or *lin-54* mutant late embryos (S5 Table). We also tested 5 Group B genes, genes not upregulated in the microarray analyses; 2 genes were significantly upregulated (S5 Table). Observing similar upregulation of genes in *lin-35* null and *lin-54* mutant late embryos suggests that the differences in number of genes upregulated in the microarray analyses are due to stage differences and that DRM target gene misregulation becomes detectable by late embryogenesis. Together with our ChIP-seq analysis, these results allow us to consider 2 possible mechanisms for how LIN-35 may function in DRM: 1) LIN-35 is essential for DRM-mediated repression, and loss of LIN-35 causes target gene upregulation or 2) LIN-35 is not essential for DRM-mediated repression, and loss of LIN-35 causes reduced chromatin occupancy by E2F-DP and MuvB, which in turn causes target gene upregulation.

### MuvB mediates DRM target gene repression

**Fig 5:**
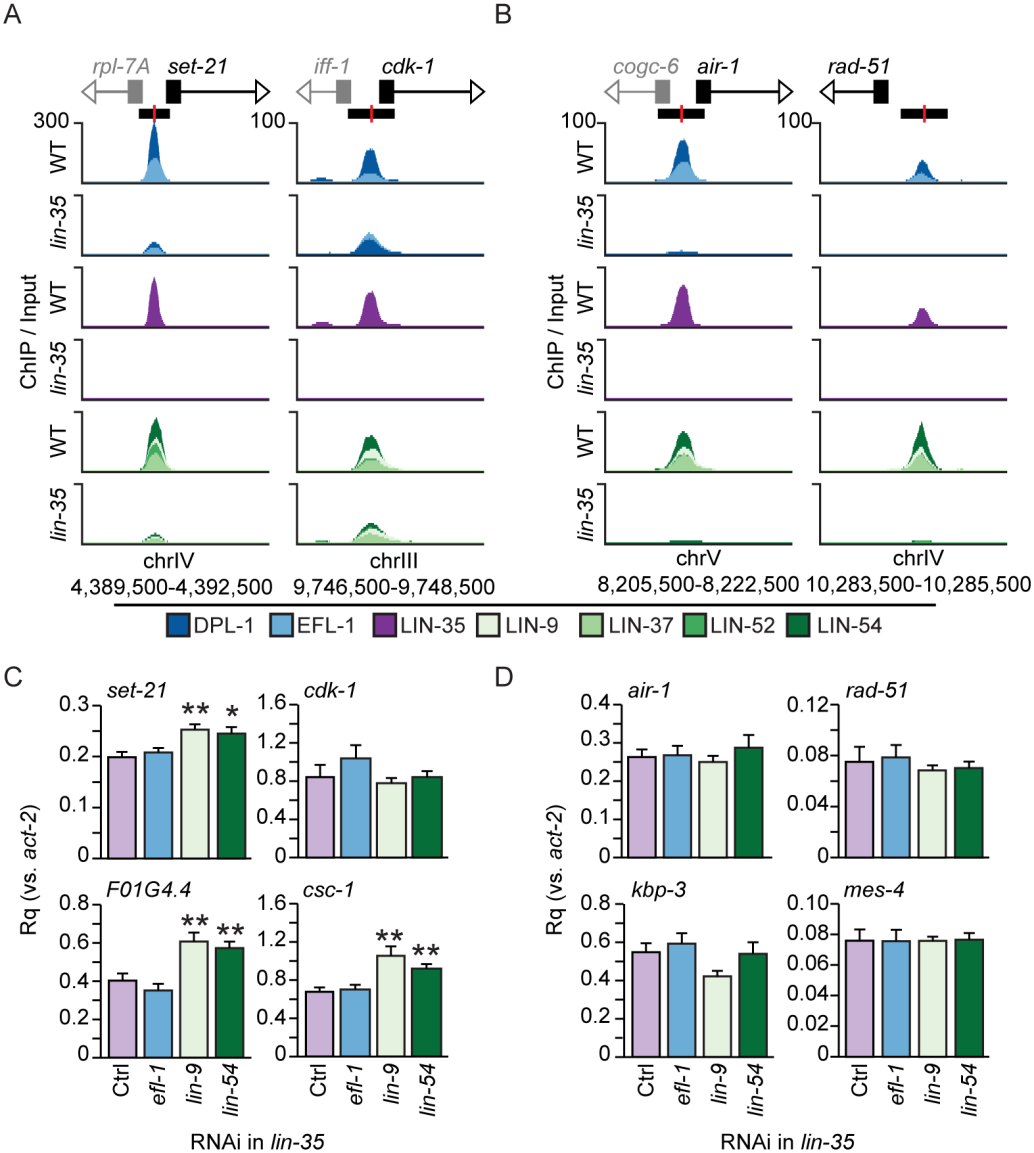
Effects of depletion of additional DRM subunits in *lin-35* null mutants. (A,B) Genomic profiles of each DRM component in wild-type (WT) and *lin-35* late embryos near (A) *set-21* and *cdk-1* and (B) *air-1 and rad-51*. E2F-DP subunits (blues), LIN-35 (purple), and MuvB subunits (greens) were overlaid on the same track. Normalized ChIP-seq enrichment values for each data track are indicated on the y-axis, and the chromosomal coordinates of the areas shown are indicated below the x-axis. Gene transcriptional start sites are indicated by black boxes, and coding strand directions are indicated by white arrowheads. Other genes in the region are similarly indicated in grey. Each DRM peak location is indicated by a black rectangle with the peak center highlighted in red. (C,D) RT-qPCR analysis of (C) *set-21*, *cdk-1*, *F01G4*.*4*, and *csc-1* and (D) *air-1*, *rad-51*, *kbp-3*, and *mes-4* following *efl-1* (blue), *lin-9* (light green), or *lin-54* (dark green) RNAi in *lin-35* null late embryos compared to empty-vector RNAi (Ctrl, purple). Expression values from 2 independent experiments each consisting of 4 biological replicates for each RNAi condition were averaged and are presented as the relative quantity (Rq) compared to *act-2*. Error bars indicate standard error of the mean, and significance was determined by a student’s T test comparing DRM subunit RNAi to Control RNAi (Ctrl) (* p-value < 0.05, ** p-value < 0.01). Additional genes are shown in S5 Table.

We reasoned that if LIN-35 is essential for repression of DRM target genes, as the current model suggests, then further disruption of E2F-DP or MuvB should not cause further upregulation of DRM target genes. Conversely, if the reduced chromatin occupancy of E2F-DP or MuvB causes upregulation of DRM target genes, then further disruption of E2F-DP or MuvB should cause further upregulation of DRM target genes whose promoters retained those components. We tested these predictions on 4 DRM target genes that retained some E2F-DP and MuvB in *lin-35* null late embryos (*set-21*, *cdk-1*, *F01G4*.*4*, and *csc-1*), and 4 DRM target genes that had no detectable E2F-DP or MuvB in *lin-35* null late embryos (*air-1*, *rad-51*, *kbp-3*, and *mes-4*) (Fig 5). We confirmed that DRM subunit binding was retained or eliminated at 4 of these respective sites by ChIP-qPCR (S3B Fig). We performed RT-qPCR analysis on *lin-35* null late embryos after two generations of RNA interference (RNAi) knockdown of *efl-1*, *lin-9*, or *lin-54*. The transcript levels of each targeted subunit were knocked down by at least 50% compared to empty vector control (S10A Fig). Knockdown of *efl-1* had no effect on transcript levels from the 8 genes tested (Fig 5C and 5D); we also confirmed a previous report [41] that in the absence of LIN-35, E2F-DP does not activate germline genes in late embryos (S10 Fig). In contrast to *efl-1*, knockdown of *lin-9* and *lin-54* caused a significant increase in transcript levels from 3 of the 4 genes where MuvB retains some observable chromatin occupancy in *lin-35* null late embryos and 0 of the 4 genes where MuvB chromatin occupancy is abolished in *lin-35* null late embryos (Fig 5C and 5D). Testing of additional genes confirmed the above findings (S5 Table). Of 24 total genes to which MuvB remains bound in *lin-35* null late embryos, 10 genes were further upregulated following knockdown of MuvB but not following knockdown of *efl-1* (S5 Table). Of 6 total genes where MuvB occupancy is lost in *lin-35* null embryos, 0 genes were further upregulated following knockdown of MuvB or *efl-1*. These results indicate that MuvB continues to repress DRM target genes in *lin-35* null embryos, strongly supporting a role for MuvB as the mediator of DRM target gene repression.

### Loss of MuvB activity in *lin-35* null worms has a catastrophic effect on fertility

**Fig 6:**
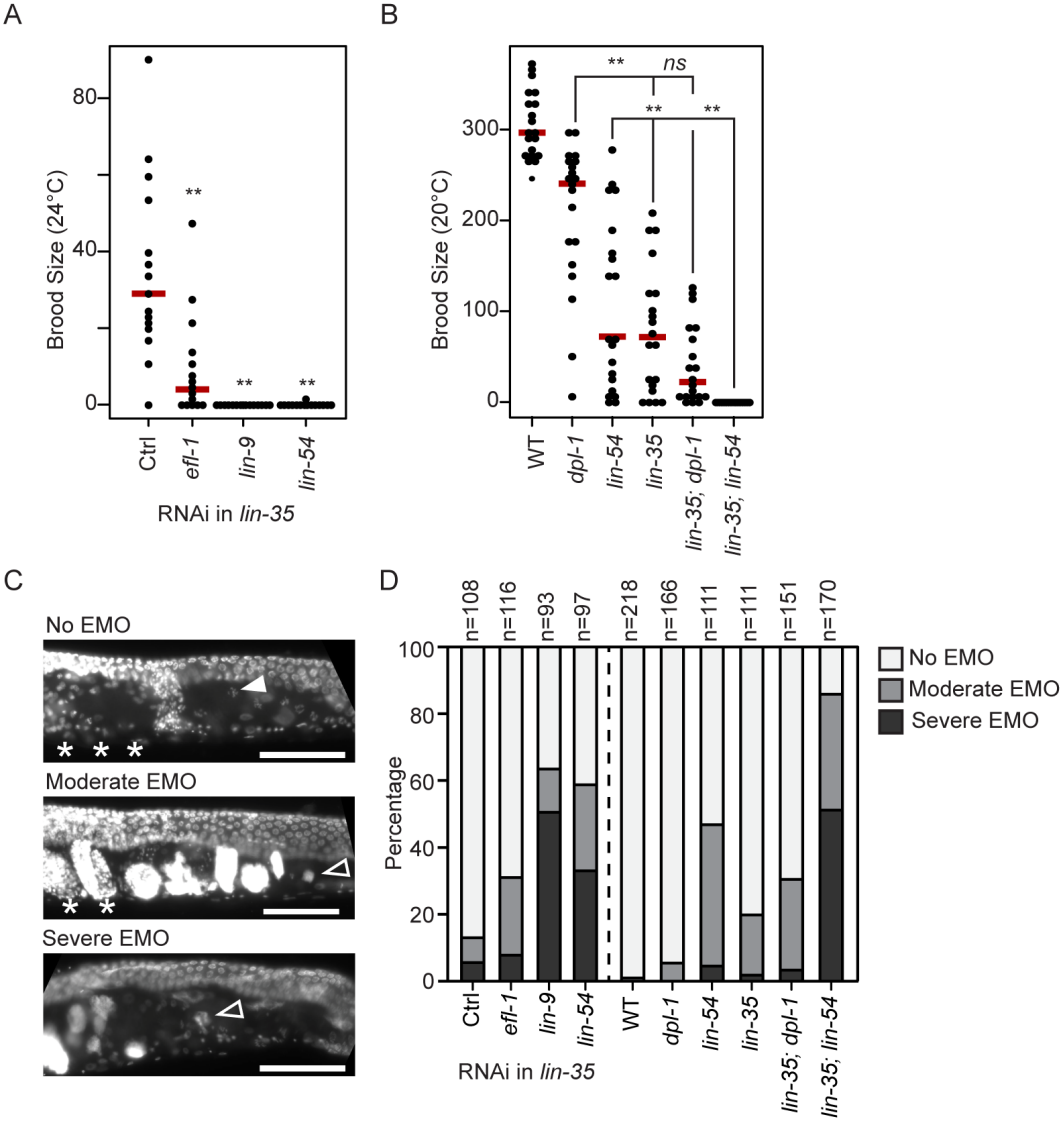
Phenotypic effects of depletion of MuvB or E2F-DP in *lin-35* null worms. (A,B) Strip chart of the brood size of (A) *lin-35* null worms following *efl-1*, *lin-9*, or *lin-54* RNAi at 24°C and (B) wild-type (WT), single DRM mutants, and double DRM mutants at 20°C. Each dot indicates the total brood of an individual worm, and a red bar indicates the median for each genotype. Significance (** p-value < 0.01) was determined by a Wilcoxon-Mann-Whitney test comparing RNAi to empty vector control (Ctrl, A) or double mutants to their respective single mutants (B). (C) DAPI staining of young adults. The filled arrowhead indicates a representative healthy oocyte, open arrowheads indicate representative endomitotic oocytes (EMO), and asterisks indicate normal-appearing embryos. No EMO: no DAPI-stained DNA masses in the oviduct, and presence of normal-appearing embryos in the uterus. Moderate EMO: presence of some DAPI-stained DNA masses in the oviduct and some normal-appearing embryos in the uterus. Severe EMO: presence of DAPI-stained DNA masses in the oviduct and no normal-appearing embryos in the uterus. Scale bar, 50 μm. (D) Normalized stacked bar chart of the EMO phenotype of Day 1 adult *lin-35* null worms with DRM subunit RNAi or double DRM mutants compared to single mutants and WT.

The analyses described above show that E2F-DP and MuvB chromatin occupancy is reduced but not eliminated in the absence of LIN-35, even though E2F-DP and MuvB protein association is completely decoupled. In our RNAi RT-qPCR analysis, we observed that knockdown of MuvB in *lin-35* null embryos resulted in further upregulation of DRM targets where MuvB retains some chromatin occupancy. While performing this experiment, we observed far fewer collected embryos in MuvB RNAi conditions compared to *efl-1* and control RNAi. Indeed, the brood size of individual *lin-35* null worms following knockdown of other DRM subunits was significantly reduced in *efl-1* RNAi and severely reduced in *lin-9* and *lin-54* RNAi compared to empty vector controls (Fig 6A). Reduction in brood size was also seen after we introduced hypomorphic alleles *dpl-1(n3643)*, which produces a truncated DPL-1 protein product [25], and *lin-54(n2231)* into the sensitized *lin-35* null background. We observed a significant decrease in brood size in *lin-35* null, *dpl-1(n3643)* hypomorphic, and *lin-54(n2231)* hypomorphic single mutants compared to WT (Fig 6B). We did not observe a significant reduction of brood size in *lin-35(n745); dpl-1(n3643)* double mutants compared to the *lin-35(n745)* single mutants (Fig 6B). In contrast, in *lin-35(n745); lin-54(n2231)* double mutants, we observed complete sterility. Together, these results indicate that further disruption of MuvB activity in *lin-35* null worms causes a severe reduction in the production of viable progeny.

Importantly, null mutations of individual E2F-DP and MuvB subunits cause sterility due to a severe EMO phenotype (production of endomitotic oocytes) [33, 36, 42], whereas the brood size reduction observed in *lin-35* null worms is associated with a moderate EMO phenotype (Fig 6C) [33]. E2F-DP null sterility is linked to its activating role in the germline, which is distinct from its role in DRM [36, 43]. However, MuvB null sterility is due to a defect in somatic sheath development [42]. We tested for EMO in *lin-35* null worms depleted of other DRM subunits. In *lin-35* null worms, RNAi knockdown of *lin-9* or *lin-54*, but not of *efl-1*, caused an increase in severe EMO (Fig 6D). Similarly, *lin-35; lin-54(n2231)* double mutants, but not *lin-35*; *dpl-1(n3643)* double mutants, displayed an increase in severe EMO (Fig 6D). We conclude that further loss of MuvB repression in *lin-35* null worms is catastrophic, reproducing the severe EMO phenotype previously observed only in null alleles that likely completely eliminate DRM activity.

## Discussion

Using genomic, genetic, and cell biological experiments on *C*. *elegans* lacking the sole pocket protein, we report evidence that MuvB mediates DRM target gene repression and that MuvB does not require LIN-35 to act as a transcriptional repressor. We demonstrate that loss of the sole *C*. *elegans* pocket protein LIN-35, which abolishes E2F-DP and MuvB association, results in a dramatic decrease in chromatin occupancy by both E2F-DP and MuvB and upregulation of many DRM target genes in late embryos. To determine how LIN-35 contributes to DRM gene repression, we depleted E2F-DP or MuvB subunits in *lin-35* null animals. Depletion of MuvB, but not E2F-DP, causes further target gene upregulation and enhances the *lin-35* null phenotype, indicating that MuvB directs repression of DRM target genes. Our study demonstrates that LIN-35 bridges E2F-DP and MuvB association, supporting their chromatin occupancy at target genes, and that MuvB functions innately as a transcriptional repressor of DRM target genes.

Studies of the mammalian DREAM DNA-binding motifs support the notion that MuvB mediates target gene repression. Most mammalian genes that are repressed in G_0_/G_1_ and activated in the later cell cycle contain a CDE, the binding motif for repressive E2F-DP, and a CHR, the binding motif for the LIN-54 subunit of MuvB [11]. Promoter analyses have shown that the CDE is not essential for DREAM binding; however, the presence of a CDE can enhance DREAM binding [12]. In contrast, the CHR is fully required for gene repression and activation at appropriate cell cycle stages [12]. Although our experimental approach does not eliminate the possibility that E2F-DP mediates repression of some target genes, taken together with the above motif analysis, our data support a model in which MuvB chromatin localization mediates DRM’s core repressive function.

Our assessment of the endomitotic oocytes (EMO) phenotype in the sensitized *lin-35* null background may provide a clue as to how *C*. *elegans* E2F-DP contributes to DRM activity. EMO can result from germline dysfunction, *e*.*g*. defects in meiosis or fertilization, or somatic dysfunction, *e*.*g*. defects in somatic sheath cell formation or function [44, 45]. EMO and sterility have previously been observed in null mutants of *dpl-1*, *efl-1*, *lin-9*, and *lin-54* [33, 36, 42, 43]. The *dpl-1* and *efl-1* null phenotype results from loss of E2F-DP’s transcriptional activation role in the germline, although some unknown somatic component is also involved [36, 43]. In contrast, the *lin-9* null phenotype results from defects in somatic sheath development [42]. With E2F-DP decoupled from MuvB in *lin-35* null worms, *efl-1* knockdown resulted in significant reduction in fertility but no enhancement of the EMO phenotype. In E2F-DP null worms, we suspect that E2F-DP loss affects MuvB function in somatic cells but only when LIN-35 is present, and that effect is overshadowed by loss of E2F-DP activating function in the germline. Thus, we speculate that E2F-DP function in DRM is to support the association of MuvB with gene promoters, with the pocket protein acting as the intermediary.

In *C*. *elegans*, loss of DRM activity leads to ectopic expression of germline genes in somatic cells [27, 28]. Interestingly, in wild-type worms LIN-35 chromatin localization is dramatically reduced in the germline compared to somatic tissues [41]. Additionally, EFL-1 and DPL-1 are known activators of the germline oogenic program [36], and by ChIP-seq they localize to more genomic regions in the adult germline than in late embryos [41]. However, a previous study found no evidence for E2F-DP adopting an activating function in the absence of LIN-35 and directly activating germline gene expression in somatic cells [41]. Our analysis also found no evidence for *C*. *elegans* E2F-DP occupying new genomic sites in *lin-35* null embryos that are normally only observed in the adult germline. Perhaps an additional event following loss of LIN-35 must occur before E2F-DP can localize to and activate a germline program. Our study further suggests that some DRM target genes require secondary regulatory factors in addition to loss of DRM for their activation. As an example, E2F-DP was recently shown to co-regulate some developmental genes with heat-shock factor (HSF) [46]. It is likely that additional regulatory networks overlap DRM functionality at certain DRM targets and coordinate their activation in specific tissues and times during development.

**Fig 7:**
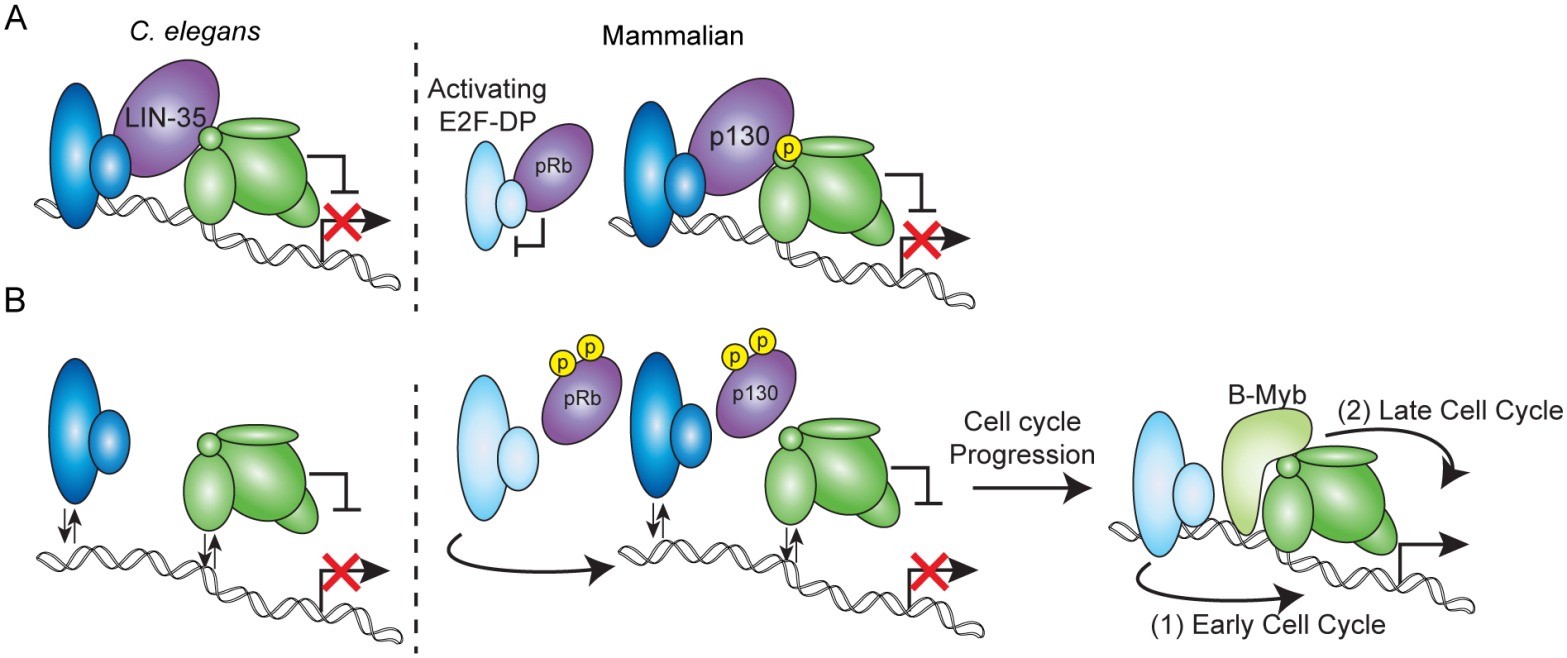
Model of DRM/DREAM function in cell cycle progression. (A) Model of the *C*. *elegans* DRM complex (left) and mammalian DREAM complex (right) activity with an intact pocket protein: E2F-DP (blue), the pocket proteins LIN-35/p130 and pRb (purple), and the 5-subunit MuvB core (green). Mammalian activating E2F-DP heterodimers (light blue) are indicated bound to pRb. Phosphorylation of LIN-52 in mammalian DREAM is based on [6]. (B) Model of the *C*. *elegans* DRM complex (left) and mammalian DREAM complex (right) activity in the absence of a pocket protein. Phosphorylation of mammalian pRb and p130 is based on [2, 7, 8]. MuvB-mediated repression of target genes is released as the cell cycle progresses, which leads to 1) activating E2F-DP heterodimers activating early cell cycle genes and 2) B-Myb association with MuvB activating late cell cycle genes [3].

Our analysis of *C*. *elegans* DRM provides insight into how the mammalian DREAM complex components maintain cellular quiescence and promote the transition into the cell cycle. Upon exit from the cell cycle, p130/p107 promotes E2F-DP and MuvB association on chromatin, stabilizing MuvB-mediated transcriptional repression of important cell cycle gene targets (Fig 7A). We speculate that the effects on DRM activity observed in *lin-35* null embryos mimic the events that immediately follow phosphorylation-mediated release of p130 from the mammalian DREAM complex, an event triggered by progression into the cell cycle (Fig 7B) [2, 7]. Loss of the pocket protein destabilizes the chromatin association of E2F-DP and MuvB, but remaining MuvB continues to repress some target genes. In mammalian cells, MuvB-mediated repression is likely overwhelmed by 1) the release of activating E2F-DP heterodimers from the retinoblastoma protein (pRb), which promotes progression into the early cell cycle [47] and 2) incorporation of MuvB into a transcriptional activation complex with B-Myb in the late cell cycle [20, 21]. Future work on how MuvB’s regulatory role is altered from repression to activation by B-Myb will help answer how precise temporal activation of cell cycle genes is achieved.

The MuvB complex’s dual transcriptional role in mammalian cells has obscured how it may contribute to mammalian DREAM-mediated gene repression. Recently, it was hypothesized that DREAM stably positions a nucleosome at the transcriptional start site of mammalian DREAM target gene promoters to prevent transcriptional activation [13]. A likely candidate for mediating DREAM-nucleosome interactions is the MuvB component RBAP48 (in *C*. *elegans*, LIN-53), a WD-repeat family protein known to bind histones when present in other complexes [24]. The RBAP48 ortholog in *Drosophila* has been shown to be required for dREAM-mediated gene repression [48]. Additionally, in *C*. *elegans*, LIN-53’s role may include facilitating deposition of the histone variant HTZ-1/H2A.Z within the body of target genes [29]. If MuvB positions nucleosomes through LIN-53/RBAP48, this would represent a novel mechanism for transcriptional repression that employs targeted non-enzymatic inhibition of transcriptional initiation.

Our demonstration of MuvB’s innate repressive role may provide clues as to how the DREAM complex components evolved to meet diverse biological needs. Phylogenetic analyses suggest that the activating E2F-DPs and pRb coevolved, diverging from their respective common ancestors, which are more similar to the repressive E2F-DPs and p130/p107 DREAM components [49, 50]. Our findings in *C*. *elegans* indicate that MuvB mediates the repressive functions of the worm DREAM complex, and can perform this activity in the absence of p130/p107 and a link to E2F-DP. Thus, if DREAM-associated gene repression represents the ancestral function of E2F-DP and pocket proteins, MuvB may have emerged prior to or coincident with E2F-DP and a pocket protein. Moreover, since *C*. *elegans* does not have a B-Myb homolog, MuvB’s repressive mechanism likely represents its ancestral function, with its activating role emerging more recently [26].

Compared to the strong tumor suppressor activity of pRb, functional redundancy in p130/p107 has obscured if and how they act as tumor suppressors [51]. Interestingly, all 3 mammalian pocket proteins are specifically targeted for degradation by the E7 oncoprotein in high-risk human papillovirus (HPV) [52, 53]. Concurrently, HPV16 E7 stimulates MMB assembly and gene activation [54]. These findings illustrate how cancer cells may drive cell cycle progression by promoting disassembly of DREAM through inactivation of p130/p107 while also coaxing MuvB into its activating function. Our results describe how the pocket protein stabilizes MuvB-mediated repression, revealing that loss of the pocket protein in cancer cells may not immediately or completely relieve MuvB repression. Cancer cell retention of the MuvB complex could allow for both oncogenic transformation through MMB-mediated activation of cell cycle genes [55] and escape from cytotoxic chemotherapy by induced reentry into quiescence through MuvB’s innate repressive activity [56]. We expect that future work on how MuvB both activates and represses its target genes will provide much-needed insights into how cancers hijack cell cycle control.

## Materials and methods

### Worm strains, culture conditions, and late embryo harvesting

All strains were cultured at 20°C using standard methods, unless otherwise noted. N2 (Bristol) was used as wild type (WT). Mutant alleles that were used in this study are listed in S6 Table. For CoIP and ChIP experiments, late stage embryos were collected by bleaching gravid worms and aging embryos for up to 3.5 hours before freezing them in liquid nitrogen.

### Co-immunoprecipitation (CoIP)

Following embryo collection, extracts were prepared and CoIP performed based on [5]. Frozen late embryos were ground using a mortar and pestle, resuspended in lysis buffer (25 mM HEPES pH 7.6, 150 mM NaCl, 1mM DTT, 1mM EDTA, 0.5 mM EGTA, 0.1% Nonidet P-40, 10% glycerol) with Complete EDTA-free protease inhibitors (Roche), and sonicated twice for 30 seconds. Lysates were clarified and precleared using Protein A Dynabeads (ThermoFisher, Waltham, MA). Protein concentrations of lysates were determined using a Qubit fluorometer (ThermoFisher). 15 μg of antibody was crosslinked to 100 μL Dynabeads using dimethyl pimelimidate in 0.2 M trimethylamine pH 8.2. Crosslinking was stopped using 0.1M Tris pH 8.0, and beads were washed with 0.1 M glycine pH 2.8 before being stored in phosphate buffered saline pH 7.2 with 0.05% Tween-20. 5 mg of protein lysate and 20 μL antibody-conjugated Dynabeads were incubated for 2 hours at 4°C. Each IP was washed with lysis buffer, eluted with 50 μL 2x SDS gel-loading sample buffer for 5 minutes at 98°C, and separated by SDS/PAGE. Antibodies used in CoIP and ChIP experiments are listed in S7 Table. Western blot analysis was performed using a 1:5,000 dilution of primary antibody and a 1:2,000 dilution of an appropriate HRP-conjugated secondary antibody. Serial western blot analysis was performed by stripping the blot with buffer containing 0.2M glycine (pH 2.2), 0.1% SDS, and 1% Tween-20 between antibody probings.

### Chromatin immunoprecipitation (ChIP)

Following embryo collection, extracts were prepared and ChIP performed based on [29]. Frozen late embryos were ground, crosslinked for 10 minutes in 1% formaldehyde, and sonicated to an average size of 250 base pairs in FA buffer (50 mM HEPES/KOH pH 7.5, 1 mM EDTA, 1% Triton X-100, 0.1% sodium deoxycholate, 150 mM NaCl). Protein concentrations of lysates were determined using a Qubit fluorometer. Lysates were precleared with Protein A Dynabeads. ChIPs were performed with 1–2 mg of extract, and 2% of the extract was set aside for an input reference control. 1–5 μg of antibody were used for ChIP-seq analysis, and 0.5 μg of antibody were used for ChIP-qPCR analysis. ChIPs were incubated overnight at 4°C with 1% sarkosyl. Protein A Dynabeads equilibrated in 20 μL FA buffer were added and incubated for 2 hours at 4°C. ChIPs were washed with the following buffers: once with FA buffer containing 1 M NaCl, once with FA buffer containing 0.5 M NaCl, once with TEL buffer (10 mM Tris-HCl pH 8.0, 0.25 M LiCl, 1% NP-40, 1% sodium deoxycholate, 1 mM EDTA), and twice with TE buffer (10 mM Tris-HCl pH 8.0 and 1 mM EDTA). 2 elutions of 50 μL elution buffer containing TE plus 1% SDS and 250 mM NaCl were incubated at 55°C. Eluted ChIP and input samples were incubated with proteinase K for 1 hour at 55°C. Crosslinks were reversed overnight at 65°C. DNA was purified by phenol-chloroform extraction and ethanol precipitation using glycogen as a carrier. Quantitative PCR was performed using SYBR green reagents on a LightCycler 480 (Roche, Basel, Switzerland) or an Applied Biosystems ViiA 7 Real-Time PCR System (ThermoFisher) using primers specific to select promoter regions, which are provided in S8 Table, and normalized against signal from a negative control region on chromosome IV.

### ChIP-seq preparation and analysis

Sequencing libraries were prepared from genomic DNA fragments (input) or those obtained after ChIP using the TruSeq ChIP Sample Prep Kit (Illumina, San Diego, CA). Amplified libraries were size selected to obtain 200–500 bp fragments using Agencourt AMPure XP beads (Beckman Coulter, Brea, CA). After verifying library fragment sizes using a 2100 Bioanalyzer (Agilent, Santa Clara, CA), sequencing was performed using the Illumina HiSeq 2000/4000 platforms. Libraries were sequenced at the Vincent J. Coates Genomics Sequencing Laboratory at the University of California, Berkeley. Sequencing reads were mapped to the ce10 reference genome using bowtie-1.1.2 [57], allowing a maximum of 2 reported alignments. ChIP-seq data were normalized to input using the signal extraction scaling (SES) method using deepTools and visualized using the UCSC genome browser [58–60]. Peak calling and data reproducibility checks were performed using the SPP peak caller and Irreproducible Discovery Rate (IDR) pipeline established by the ENCODE project [61, 62]. Peak overlaps and gene mapping were processed using HOMER [63], with gene transcriptional start sites (TSS) and transcriptional terminal sites (TTS) based on RefSeq annotations. Differential binding analysis was performed using the DiffBind package in Bioconductor (www.bioconductor.org) and the R statistical programming language [64]. Motif analysis was performed *de novo* using the MEME suite [32], or overlapping known motifs using HOMER and phastCons [65]. Motif sequence logos were generated using WebLogo [66]. Important quality control metrics are provided in S9 Table.

### RNA interference and transcript analysis

Bacteria from the Ahringer RNAi feeding library [67] expressing dsRNA against *efl-1*, *lin-9*, and *lin-54* were sequence-verified and fed to *lin-35(n745)* worms. Progeny from gravid worms grown in liquid media were synchronized as L1 larvae and fed RNAi on NGM plates containing 1 mM IPTG and ampicillin. RNAi feeding was administered for 2 generations at 24°C, after which late embryos from 2^nd^ generation gravid adults were collected in Trizol for RNA isolation and transcript analysis. A total of 1.5 μg RNA was DNAse treated and reverse transcribed using the High Capacity cDNA Kit (Applied Biosystems, Foster City, CA). qPCR was performed using SYBR green reagents on a LightCycler 480 (Roche) or an Applied Biosystems ViiA 7 Real-Time PCR System (ThermoFisher) using primers specific to a select gene set, which are provided in S10 Table. The relative quantity of experimental transcripts was calculated with *act-2* as the control gene using the ΔCt method with efficiency correction. Statistical analysis of genome-wide expression data was performed using R, using the Quantile normalization and Robust Multichip Average (RMA) algorithm in the affy package from Bioconductor [68, 69]. To determine differential expression, moderated T Statistics were applied using the limma package, using *q*-value < 0.05 and fold change > 1.5 as the significance thresholds [70]. For EMO and brood size scoring, 2^nd^ generation RNAi-fed L4 larvae were aged overnight and scored.

### *C*. *elegans* phenotype scoring

For endomitotic oocyte (EMO) scoring, L4 larvae were aged overnight, fixed using Carnoy’s solution, stained with the DNA intercalator DAPI, and scored blind. The definitions of severe EMO, moderate EMO, and no Emo are in the Fig 6 legend. Images were acquired using a Zeiss Axioskop (Oberkochen, Germany) and processed using Image J [71]. For brood size analyses, individuals were cloned to fresh plates every 24 hours starting at the L4 larval stage and all progeny were counted.

## Supporting information

S1 Fig All DRM subunits co-occupy similar sites with no appreciable independent chromatin association of individual subunits or sub-complexes. (A) UpSet visualization of DRM subunit peak overlaps. Black filled circles indicate the factor(s) present within an intersection category. Intersection categories consisting of less than 20 represented peaks were omitted. (B) Heatmap of normalized ChIP-seq profiles of pooled replicates for each DRM subunit across all identified peaks. High confidence DRM peaks (identified in Fig 1) are separated from the peaks that did not pass the overlap requirements (remaining DRM peaks). ChIP-seq signal appears consistently stronger in the high confidence DRM peaks when compared to the remaining DRM peaks. Even though many peaks do not pass the overlap requirements, the occupancy observed for each DRM subunit appears similar at all peak regions.
(TIF)

S2 Fig LIN-35 mediates the association of E2F-DP and MuvB. Full western blots from 2 co-immunoprecipitation experiments performed on biological replicate late embryo lysates. Proteins bound (B) and unbound (UB) by EFL-1, LIN-37, or control IgG immunoprecipitation were separated by SDS/PAGE, and western blot analysis was performed using the antibodies indicated in the bottom right corner. 5% of Input was included. Arrows indicate protein bands presented in main Fig 2. Asterisks indicate bands that carried over from a previous blot.
(TIF)

S3 Fig Chromatin occupancy of DRM subunits decreases genome-wide in *lin-35* null compared to wild-type late embryos. (A) Scatter plot of normalized ChIP-seq reads in *lin-35* vs. wild type (WT) for each DRM subunit for Class I (dark) and Class II (light) peaks, as described in the Fig 3 legend. Each point indicates the WT (x-axis) and *lin-35* null (y-axis) read density of pooled replicates within a single wild-type DRM peak from E2F/DP (blue), LIN-35 (purple), and MuvB (green) subunit ChIP-seq. Dotted lines indicate the slope expected if DRM occupancy is equivalent in WT and *lin-35* ChIP-seq. (B) ChIP-qPCR of 5 DRM subunits at 8 Class I peaks and 4 Class II peaks. H3K4me3 ChIP was included as a positive control. Signals are presented as the log_2_ fold enrichment of the ChIP signal for each region vs. a negative control region (a non-coding region of chromosome IV). Error bars indicate standard error of the mean. Significance was determined by a student’s T test between subunit ChIP values in WT versus *lin-35* (* p-value < 0.05, ** p-value < 0.01) or between subunit versus IgG ChIP values in *lin-35* († p-value < 0.05, ‡ p-value < 0.01). The chromatin occupancy of the majority of DRM subunits significantly decreased at all sites tested. Additionally, the majority of DRM subunits retained significant chromatin enrichment over IgG in *lin-35* null embryos at 9 of the 12 sites tested.
(TIF)

S4 Fig In 2 of the 3 *lin-35* null extracts, DRM subunit ChIP-seq signal is dramatically decreased compared to wild type. Box plots of normalized read counts in DRM peaks in wild-type (WT) versus *lin-35* null replicates for each DRM subunit. Box plots show the median (black bar) with each box extending from the 25^th^ to 75^th^ percentile. Extended whiskers indicate the 2.5^th^ and 97.5^th^ percentile, and wedges indicate the 95% confidence interval for the medians. Outliers were removed from the graphs. Signal from the 2^nd^ *lin-35* null ChIP-seq replicate appears to be abnormally elevated compared to the 1^st^ and 3^rd^ replicates.
(TIF)

S5 Fig DRM binds upstream of several DRM subunit genes, and loss of DRM function causes upregulation of several DRM subunit transcripts but not of the corresponding proteins. (A) Genomic profiles of each DRM component in wild-type (WT) and *lin-35* late embryos near DRM subunit genes. E2F-DP subunits (blues), LIN-35 (purple), and MuvB subunits (greens) were overlaid on the same track. Normalized ChIP-seq enrichment values for each data track are indicated on the y-axis, and the chromosomal coordinates of the areas shown are indicated below the x-axis. Gene transcriptional start sites are indicated by black boxes, coding strand directions are indicated by white arrowheads, and gene transcriptional termination sites are indicated by black arrowheads. Other genes in the region are similarly indicated in grey. Each DRM peak location is indicated by a black rectangle with the peak center highlighted in red. Of the 8 DRM genes, only *lin-37* and *lin-52* are not targeted by a promoter-bound DRM peak. (B) RT-qPCR analysis comparing transcript levels of DRM subunit genes in *lin-35(n745)* (purple) and *lin-54(n2231)* (green) late embryos to the level in WT (white) late embryos, presented as the relative quantity (Rq) compared to *act-2*. Error bars indicate standard error of the mean, and significance was determined by a student’s T test between transcript levels in mutant vs WT (* p-value < 0.05, ** p-value < 0.01). 7 of the 8 DRM genes were upregulated in *lin-35* and/or *lin-54* mutants. *lin-35* transcripts in *lin-35* null embryos were significantly decreased compared to WT, consistent with the W151Stop* mutation leading to nonsense-mediated decay of the transcript. (C) Western blot analysis of 2 biological replicates of WT and *lin-35* lysates performed using the antibodies indicated on the right. Alpha-tubulin served as a loading control. (D) ChIP-qPCR of DRM subunits at the *mes-2*, *cdk-1*, and *pcn-1* gene promoters in L1 larvae. Signals are presented as the log_2_ fold enrichment of the ChIP signal for each region vs. a negative control region (a non-coding region of chromosome IV). Error bars indicate standard error of the mean. Significance was determined by a student’s T test between subunit ChIP values in WT versus *lin-35* (* p-value < 0.05, ** p-value < 0.01) or between subunit versus IgG ChIP values in *lin-35* († p-value < 0.05, ‡ p-value < 0.01).
(TIF)

S6 Fig Identification of conserved CDE and CHR motifs in DRM peak regions. (A,B) Enrichment of phylogenetically conserved (phasCons > 0.7) CDE-like sequences (IUPAC code: BSSSSS, allowing 1 mismatch) and CHR-like sequences (IUPAC code: TTYRAA) in DRM peak regions (1418 regions) compared to background promoters of all non-overlapping and non-DRM target genes (16,471 background regions). Black bars and bolded sequences indicate significant enrichment over background using a hypergeometric distribution (p-value < 1e-5). Not all potential CDE sequences are shown. (C,D) Motif enrichment in Class I versus Class II DRM peaks and in HOT versus not HOT DRM peaks using a chi-squared test (p < 0.05). Although we observed enrichment of the *de novo* motif in Class I peaks compared to Class II peaks, the enrichment was lost when we tested conserved CDE and/or CHR motifs. This result suggests that the presence of a DRM motif does not distinguish the two peak classes. Conserved CDE and CHR motifs were observed in HOT DRM peaks, suggesting that many DRM peaks in HOT regions are true DRM binding sites.
(TIF)

S7 Fig In the absence of LIN-35, E2F-DP and MuvB subunits remain confined to wild-type DRM peak regions. (A,B) Venn diagrams showing total peak overlaps for each E2F-DP subunit (A, blue) and each MuvB subunit (B, green) in *lin-35* compared to wild-type (WT) ChIP-seq. The number of subunit peaks observed only in WT (i), in both WT and *lin-35* (ii), and only in *lin-35* (iii) are indicated. Total overlaps of subunit peaks observed only in *lin-35* (iii) for E2F-DP (A) or MuvB (B) are indicated in the 2^nd^ venn diagram (grey). (C) Heatmap of normalized ChIP-seq profiles of pooled replicates for each DRM subunit. Plotted regions for each subunit are separated based on whether the peak was observed only in wild type (WT) (i), observed in both WT and *lin-35* (ii), or observed only in *lin-35* (iii). Many "*lin-35* only" peaks reside near DRM binding sites already observed in WT.
(TIF)

S8 Fig Upregulation of DRM target genes in DRM mutants appears stage-dependent. (A) Venn diagrams showing DRM target genes that are upregulated based on microarray analysis from [37] (*lin-35* early embryo (EE) and L1, light and dark purple) and [33] (*lin-54* mixed-stage embryo (ME), green) (Group A genes). (B) Box plots of wild-type (WT) or mutant (*lin-35* or *lin-54)* log_2_ average expression values of Upregulated DRM targets (Group A), Not Upregulated DRM Targets (Group B), and All Genes. Staging of the samples for each experimental group is indicated: early embryos (EE), mixed-stage embryos (ME), and L1 larvae (L1). Box plots show the median (black bar) with each box extending from the 25^th^ to 75^th^ percentile. Extended whiskers indicate the 2.5^th^ and 97.5^th^ percentile, and wedges indicate the 95% confidence interval for the medians. Outliers were removed from the graphs. Significance (* p-value < 0.05, ** p-value < 0.01) was determined by a Wilcoxon-Mann-Whitney test. These results indicate that detectable upregulation reflects a drop in the average expression of DRM target genes in WT over developmental time, not an increase in the average expression of DRM target genes in mutants. This effect is also observed in Group B genes but the repression is not as dramatic. We speculate that Group A genes are progressively more repressed by DRM over developmental time; DRM dysfunction manifests as a failure in this repression and hence gene upregulation as development progresses. In contrast, Group B genes may include genes that 1) DRM does not fully repress or 2) are activated intermittently as development progresses. These findings suggest that detection of DRM target gene upregulation is dependent on developmental stage because wild-type DRM requires developmental time to establish repression of gene targets.
(TIF)

S9 Fig Classification of DRM Target genes by GO enrichment analysis. (A) REViGO gene ontology (GO) visualization of terms, which helped identify informative GO terms, enriched in Group A targets (left) vs. Group B targets (right), as described in Fig 5. GO terms are clustered on the x-y plane based on semantic similarity measures, with the size of each bubble indicating the GO term frequency and the color indicating the Benjamini adjusted p-value. Representative GO terms are highlighted and labeled. (B,C) Bar chart plotting the –log_10_ Benjamini adjusted p-value of selected GO term enrichment observed in (B) DRM targets marked by Class I peaks vs. Class II peaks, and (C) DRM targets marked by HOT regions vs. not HOT regions.
(TIF)

S10 Fig Knockdown efficiency of DRM subunits in *lin-35* null embryos. (A) RT-qPCR quantification of the effectiveness of RNAi depletion of *efl-1* (blue), *lin-9* (light green), and *lin-54* (dark green) transcript levels in *lin-35* null (*lin-35(n745))* late embryos compared to empty-vector (Ctrl, purple) RNAi. (B) Genomic profiles of each DRM component in wild-type (WT) and *lin-35* null late embryos at *rme-2* and *mex-5*. E2F/DP subunits (blues), LIN-35 (purple) and MuvB subunits (greens) were overlaid on the same track. Normalized ChIP-seq enrichment values for each data track are indicated on the y-axis, and the chromosomal coordinates of the areas shown are indicated below the x-axis. Gene transcriptional start sites are indicated by black boxes, and coding strand directions are indicated by white arrowheads. (C) RT-qPCR analysis of *rme-2* and *mex-5* following *efl-1* (blue), *lin-9* (light green), and *lin-54* (dark green) RNAi in *lin-35* null late embryos as compared to empty-vector (Ctrl, purple). Expression values from 2 independent experiments each consisting of 4 biological replicates for each RNAi condition were averaged and presented as the relative quantity (Rq) compared to *act-2*. Error bars indicate standard error of the mean.
(TIF)

S1 Table Wild-type DRM peak regions. (XLSX)

S2 Table *lin-35(n745)* DRM peak regions. (XLSX)

S3 Table Expression analysis of DRM target genes. (XLSX)

S4 Table DAVID GO term enrichment. (XLSX)

S5 Table RT-qPCR analyses. (TIF)

S6 Table Strain information. (TIF)

S7 Table Antibody information. (TIF)

S8 Table ChIP-qPCR primers. (TIF)

S9 Table ChIP-seq data information. (TIF)

S10 Table RT-qPCR primers. (TIF)
