# Supplementary figures and images for: Loss of the Caenorhabditis elegans pocket protein LIN-35 reveals MuvB's innate function as the repressor of DREAM target genes
Source: PLoS Genet. 2017 Nov 1;13(11):e1007088. doi: 10.1371/journal.pgen.1007088 (PMC5683655; doi:10.1371/journal.pgen.1007088)

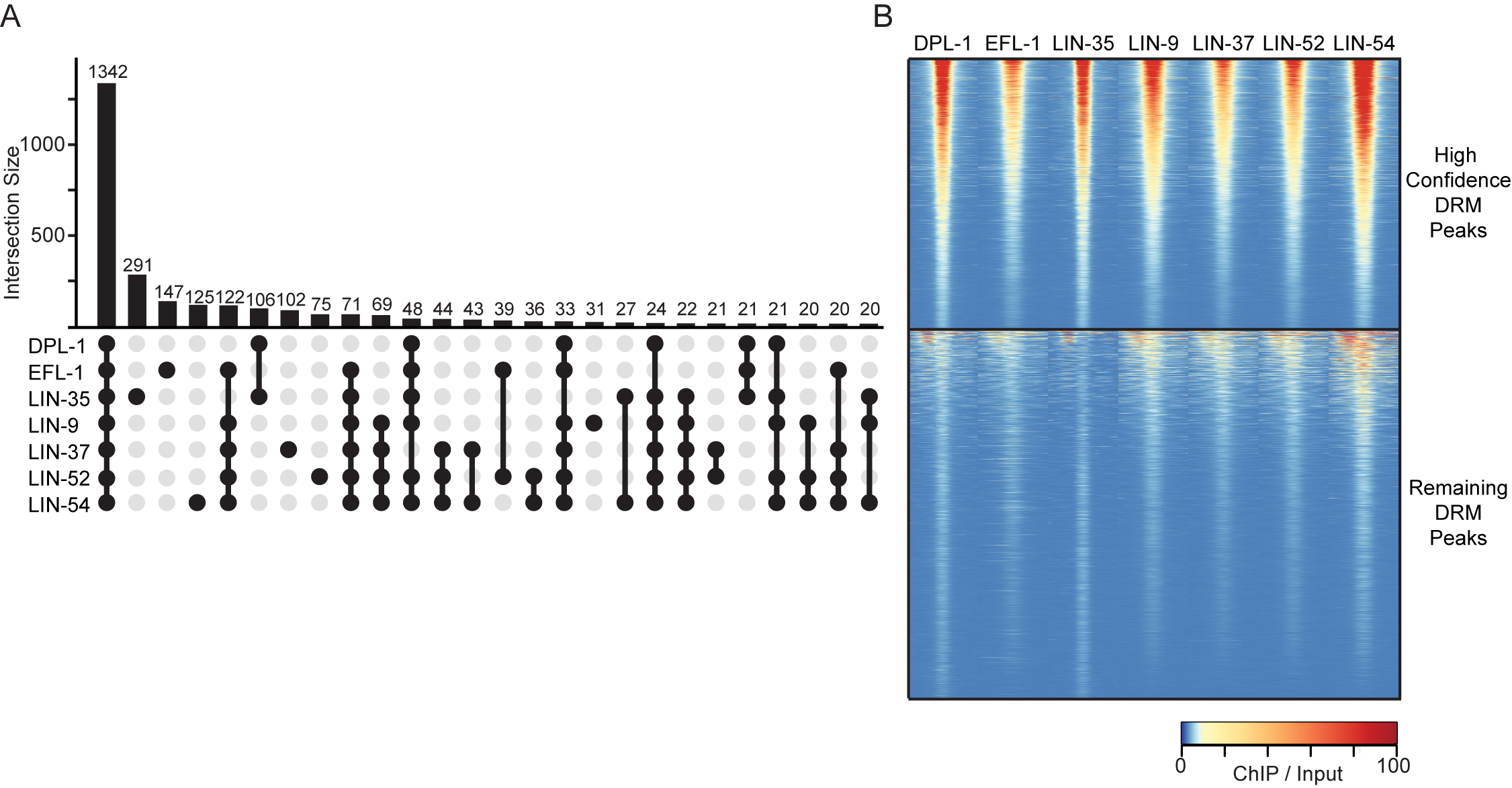

Supplement: S1 Fig — (A) UpSet visualization of DRM subunit peak overlaps. Black filled circles indicate the factor(s) present within an intersection category. Intersection categories consisting of less than 20 represented peaks were omitted. (B) Heatmap of normalized ChIP-seq profiles of pooled replicates for each DRM subunit across all identified peaks. High confidence DRM peaks (identified in Fig 1) are separated from the peaks that did not pass the overlap requirements (remaining DRM peaks). ChIP-seq signal appears consistently stronger in the high confidence DRM peaks when compared to the remaining DRM peaks. Even though many peaks do not pass the overlap requirements, the occupancy observed for each DRM subunit appears similar at all peak regions. (TIF) [file pgen.1007088.s001.tif]

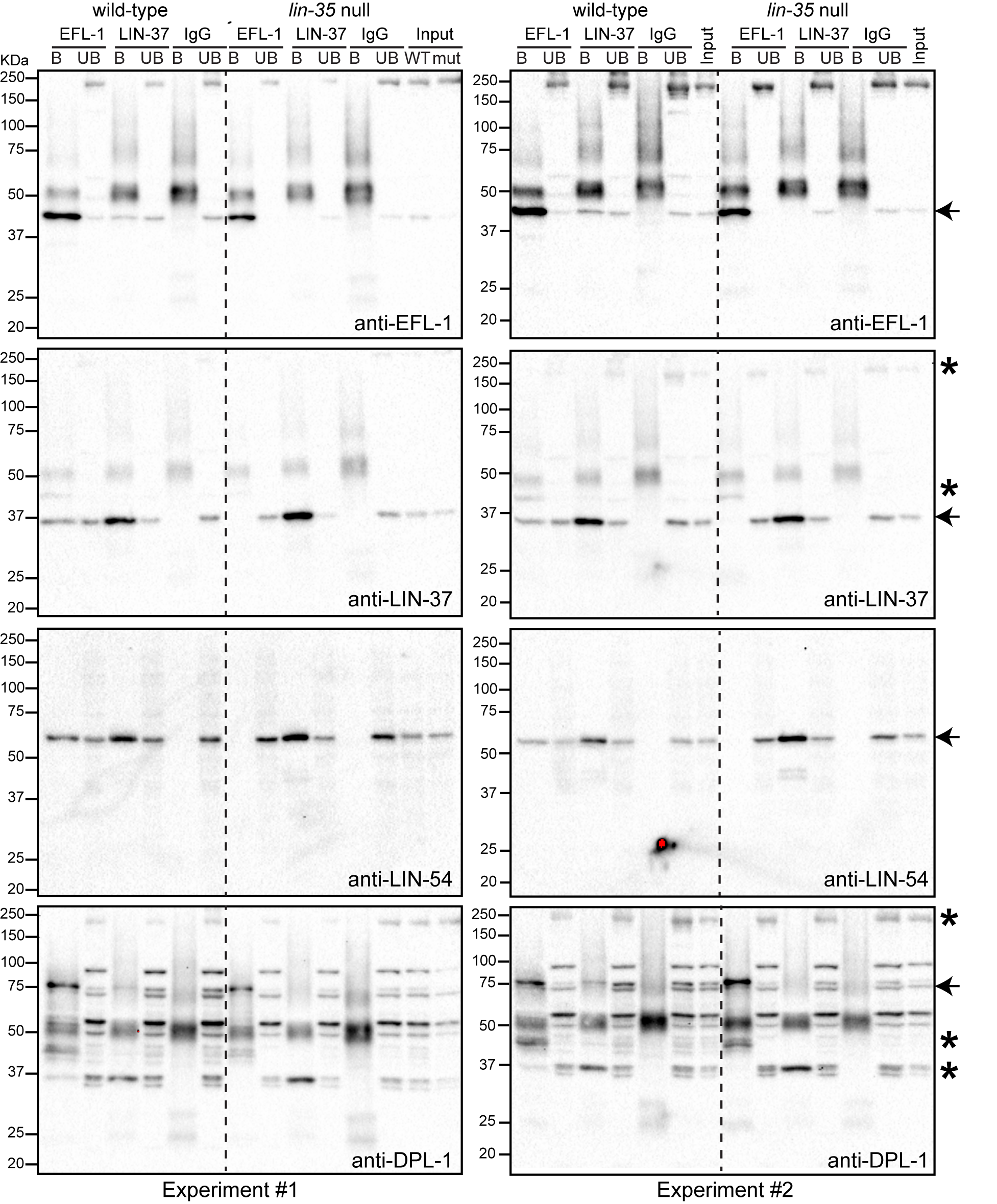

Supplement: S2 Fig — Full western blots from 2 co-immunoprecipitation experiments performed on biological replicate late embryo lysates. Proteins bound (B) and unbound (UB) by EFL-1, LIN-37, or control IgG immunoprecipitation were separated by SDS/PAGE, and western blot analysis was performed using the antibodies indicated in the bottom right corner. 5% of Input was included. Arrows indicate protein bands presented in main Fig 2. Asterisks indicate bands that carried over from a previous blot. (TIF) [file pgen.1007088.s002.tif]

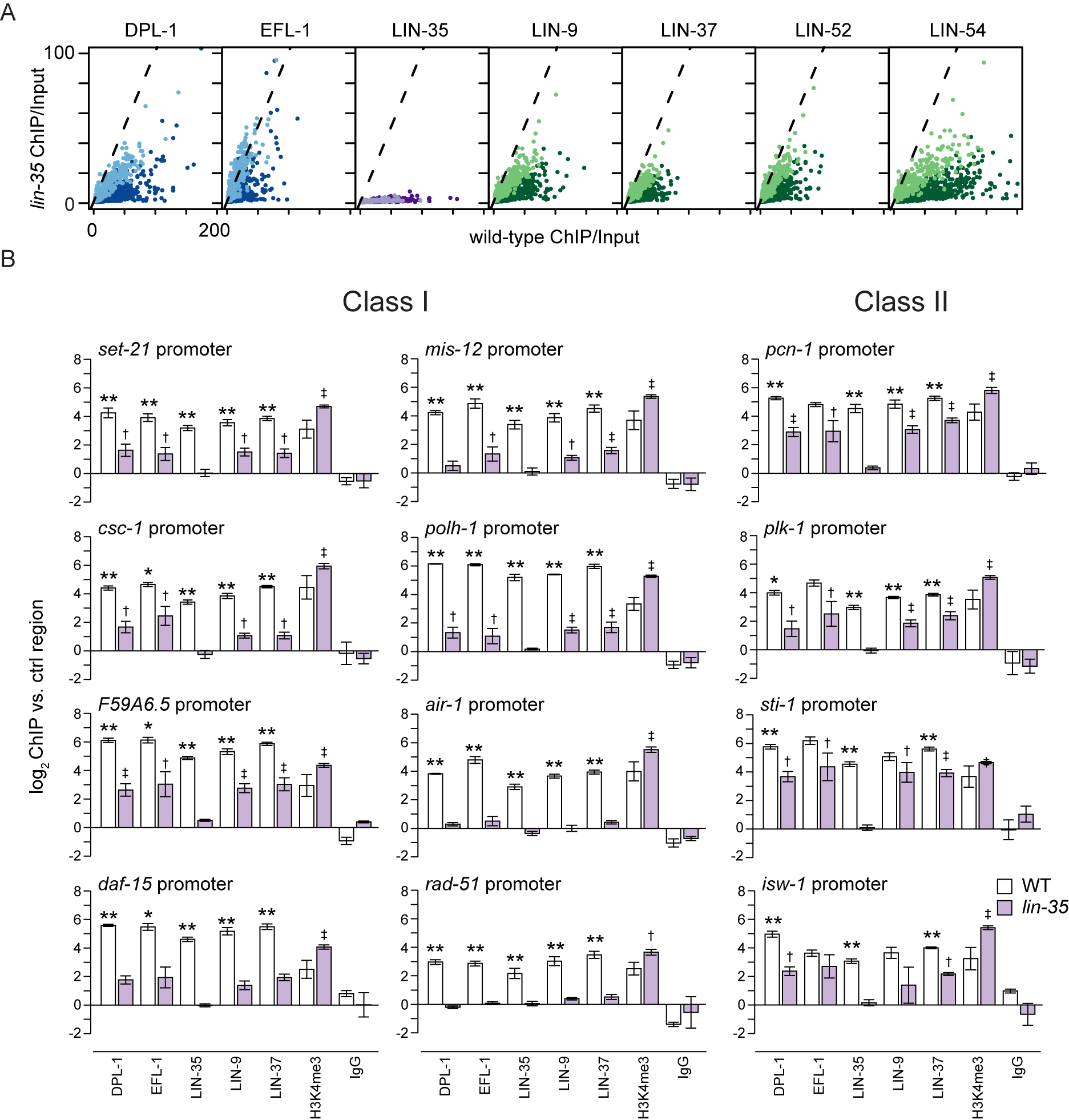

Supplement: S3 Fig — (A) Scatter plot of normalized ChIP-seq reads in lin-35 vs. wild type (WT) for each DRM subunit for Class I (dark) and Class II (light) peaks, as described in the Fig 3 legend. Each point indicates the WT (x-axis) and lin-35 null (y-axis) read density of pooled replicates within a single wild-type DRM peak from E2F/DP (blue), LIN-35 (purple), and MuvB (green) subunit ChIP-seq. Dotted lines indicate the slope expected if DRM occupancy is equivalent in WT and lin-35 ChIP-seq. (B) ChIP-qPCR of 5 DRM subunits at 8 Class I peaks and 4 Class II peaks. H3K4me3 ChIP was included as a positive control. Signals are presented as the log2 fold enrichment of the ChIP signal for each region vs. a negative control region (a non-coding region of chromosome IV). Error bars indicate standard error of the mean. Significance was determined by a student’s T test between subunit ChIP values in WT versus lin-35 (* p-value < 0.05, ** p-value < 0.01) or between subunit versus IgG ChIP values in lin-35 († p-value < 0.05, ‡ p-value < 0.01). The chromatin occupancy of the majority of DRM subunits significantly decreased at all sites tested. Additionally, the majority of DRM subunits retained significant chromatin enrichment over IgG in lin-35 null embryos at 9 of the 12 sites tested. (TIF) [file pgen.1007088.s003.tif]

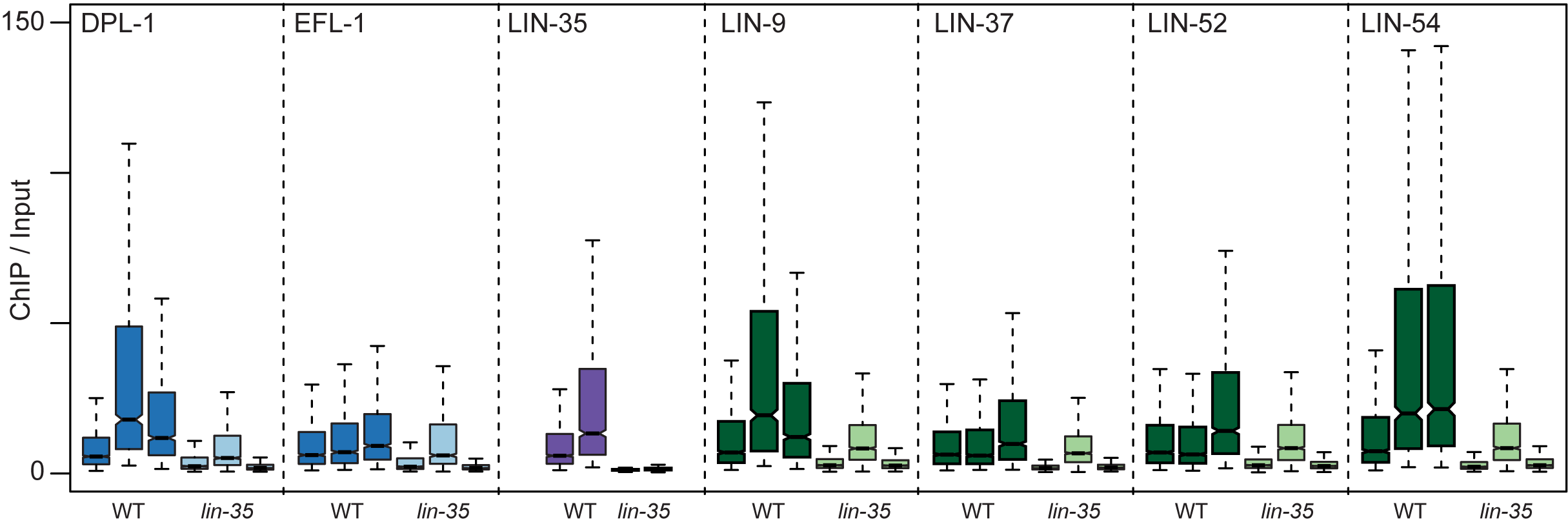

Supplement: S4 Fig — Box plots of normalized read counts in DRM peaks in wild-type (WT) versus lin-35 null replicates for each DRM subunit. Box plots show the median (black bar) with each box extending from the 25th to 75th percentile. Extended whiskers indicate the 2.5th and 97.5th percentile, and wedges indicate the 95% confidence interval for the medians. Outliers were removed from the graphs. Signal from the 2nd lin-35 null ChIP-seq replicate appears to be abnormally elevated compared to the 1st and 3rd replicates. (TIF) [file pgen.1007088.s004.tif]

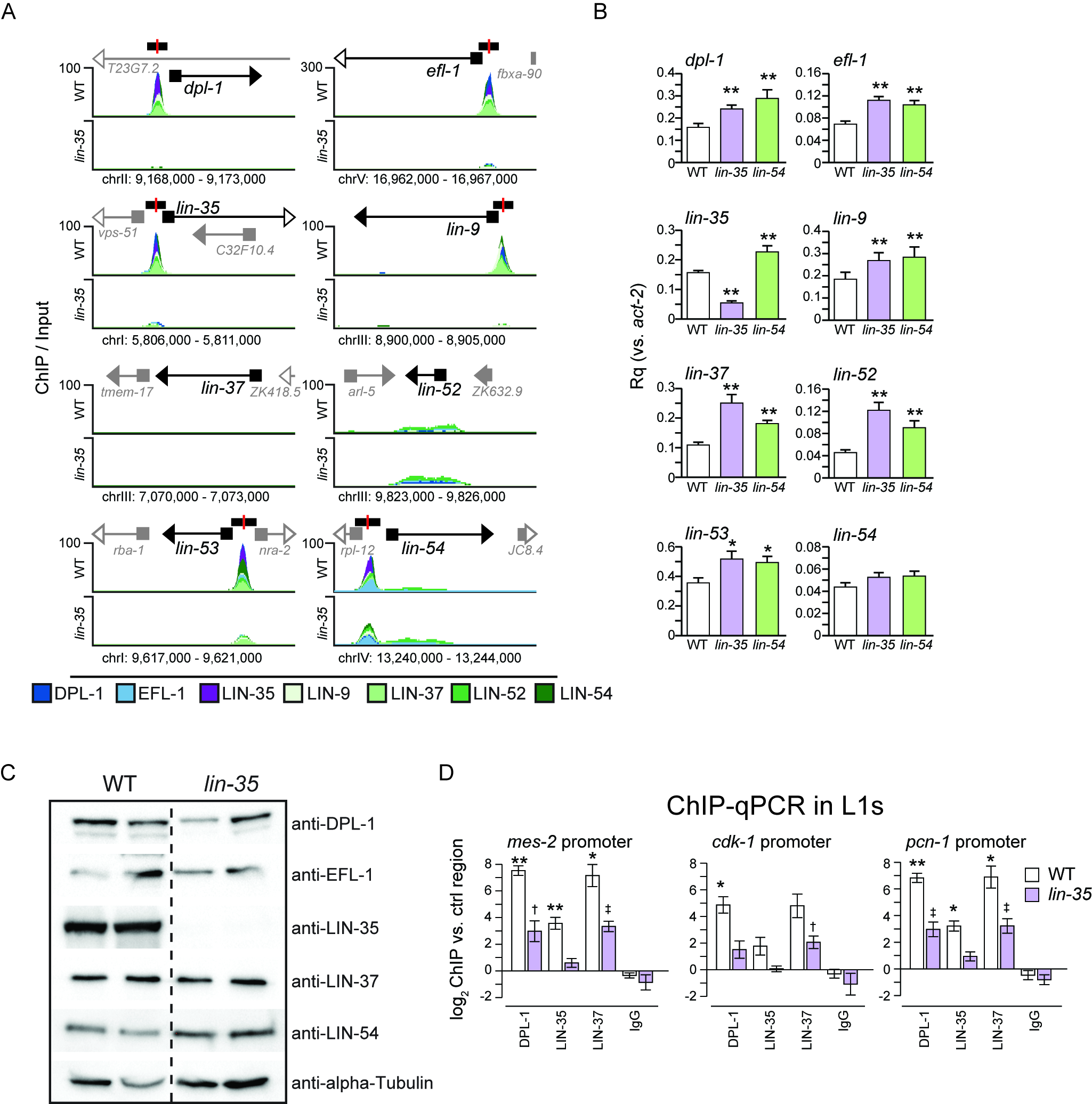

Supplement: S5 Fig — (A) Genomic profiles of each DRM component in wild-type (WT) and lin-35 late embryos near DRM subunit genes. E2F-DP subunits (blues), LIN-35 (purple), and MuvB subunits (greens) were overlaid on the same track. Normalized ChIP-seq enrichment values for each data track are indicated on the y-axis, and the chromosomal coordinates of the areas shown are indicated below the x-axis. Gene transcriptional start sites are indicated by black boxes, coding strand directions are indicated by white arrowheads, and gene transcriptional termination sites are indicated by black arrowheads. Other genes in the region are similarly indicated in grey. Each DRM peak location is indicated by a black rectangle with the peak center highlighted in red. Of the 8 DRM genes, only lin-37 and lin-52 are not targeted by a promoter-bound DRM peak. (B) RT-qPCR analysis comparing transcript levels of DRM subunit genes in lin-35(n745) (purple) and lin-54(n2231) (green) late embryos to the level in WT (white) late embryos, presented as the relative quantity (Rq) compared to act-2. Error bars indicate standard error of the mean, and significance was determined by a student’s T test between transcript levels in mutant vs WT (* p-value < 0.05, ** p-value < 0.01). 7 of the 8 DRM genes were upregulated in lin-35 and/or lin-54 mutants. lin-35 transcripts in lin-35 null embryos were significantly decreased compared to WT, consistent with the W151Stop* mutation leading to nonsense-mediated decay of the transcript. (C) Western blot analysis of 2 biological replicates of WT and lin-35 lysates performed using the antibodies indicated on the right. Alpha-tubulin served as a loading control. (D) ChIP-qPCR of DRM subunits at the mes-2, cdk-1, and pcn-1 gene promoters in L1 larvae. Signals are presented as the log2 fold enrichment of the ChIP signal for each region vs. a negative control region (a non-coding region of chromosome IV). Error bars indicate standard error of the mean. Significance was determined by a s [file pgen.1007088.s005.tif]

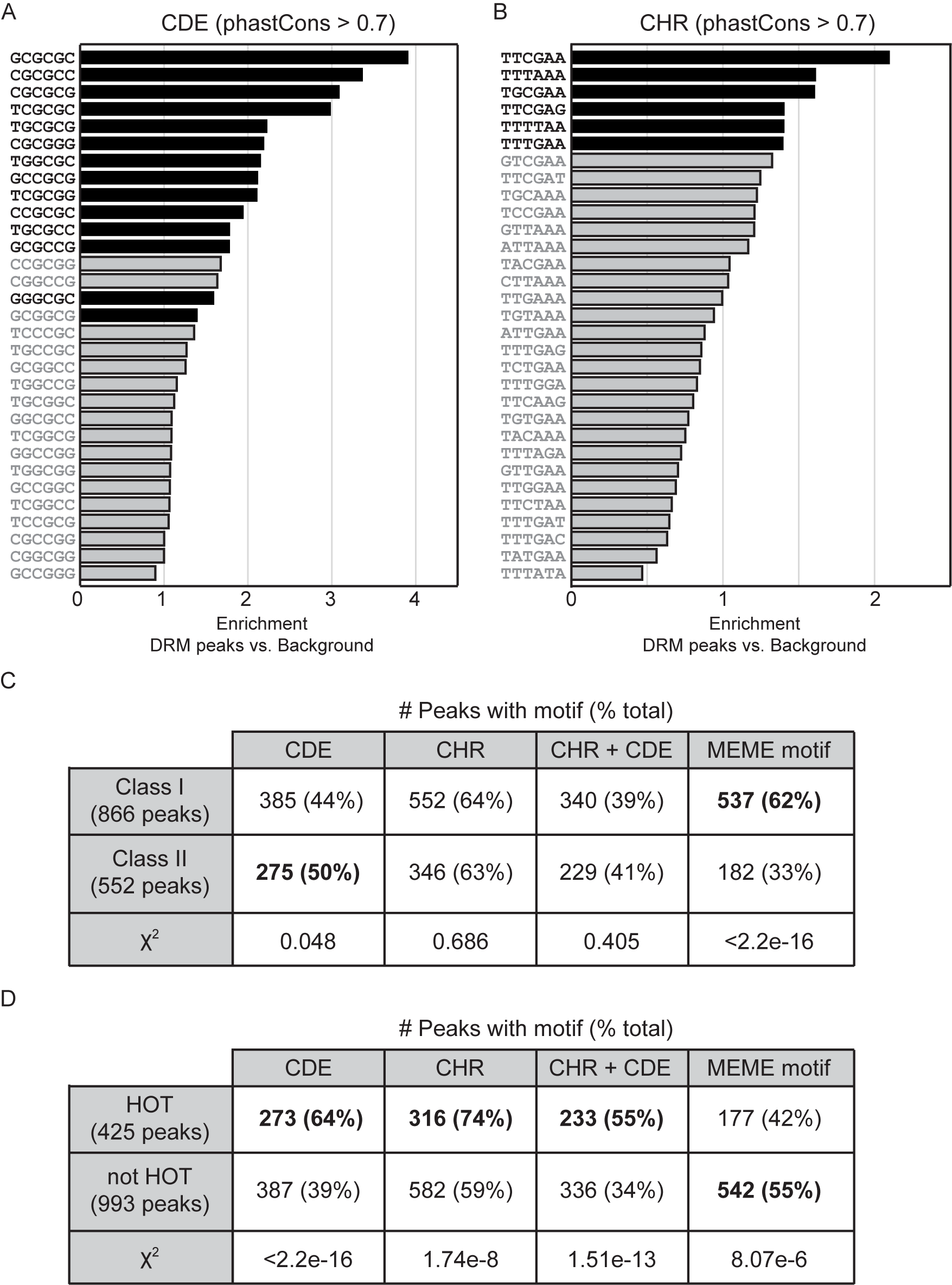

Supplement: S6 Fig — (A,B) Enrichment of phylogenetically conserved (phasCons > 0.7) CDE-like sequences (IUPAC code: BSSSSS, allowing 1 mismatch) and CHR-like sequences (IUPAC code: TTYRAA) in DRM peak regions (1418 regions) compared to background promoters of all non-overlapping and non-DRM target genes (16,471 background regions). Black bars and bolded sequences indicate significant enrichment over background using a hypergeometric distribution (p-value < 1e-5). Not all potential CDE sequences are shown. (C,D) Motif enrichment in Class I versus Class II DRM peaks and in HOT versus not HOT DRM peaks using a chi-squared test (p < 0.05). Although we observed enrichment of the de novo motif in Class I peaks compared to Class II peaks, the enrichment was lost when we tested conserved CDE and/or CHR motifs. This result suggests that the presence of a DRM motif does not distinguish the two peak classes. Conserved CDE and CHR motifs were observed in HOT DRM peaks, suggesting that many DRM peaks in HOT regions are true DRM binding sites. (TIF) [file pgen.1007088.s006.tif]

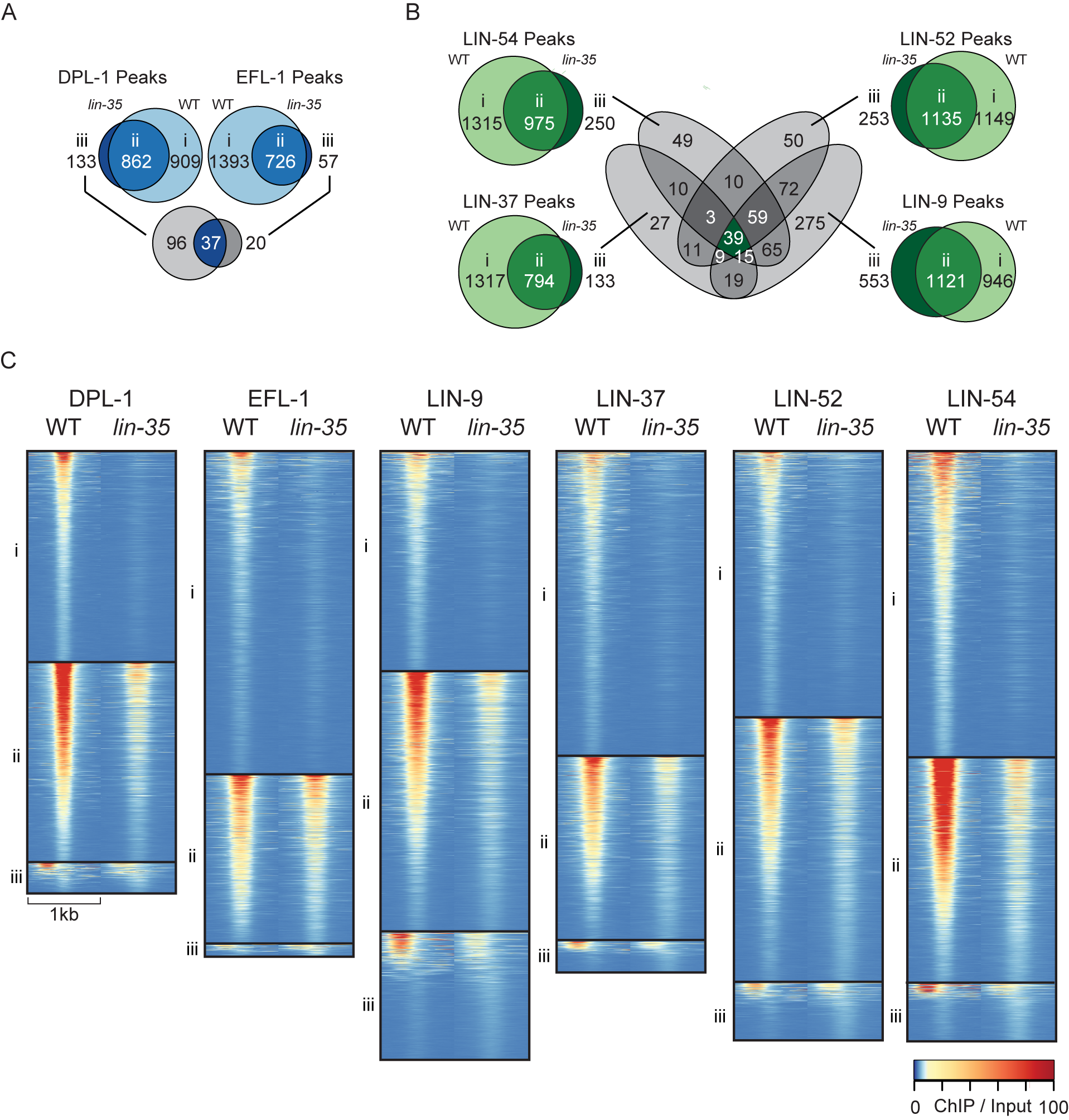

Supplement: S7 Fig — (A,B) Venn diagrams showing total peak overlaps for each E2F-DP subunit (A, blue) and each MuvB subunit (B, green) in lin-35 compared to wild-type (WT) ChIP-seq. The number of subunit peaks observed only in WT (i), in both WT and lin-35 (ii), and only in lin-35 (iii) are indicated. Total overlaps of subunit peaks observed only in lin-35 (iii) for E2F-DP (A) or MuvB (B) are indicated in the 2nd venn diagram (grey). (C) Heatmap of normalized ChIP-seq profiles of pooled replicates for each DRM subunit. Plotted regions for each subunit are separated based on whether the peak was observed only in wild type (WT) (i), observed in both WT and lin-35 (ii), or observed only in lin-35 (iii). Many "lin-35 only" peaks reside near DRM binding sites already observed in WT. (TIF) [file pgen.1007088.s007.tif]

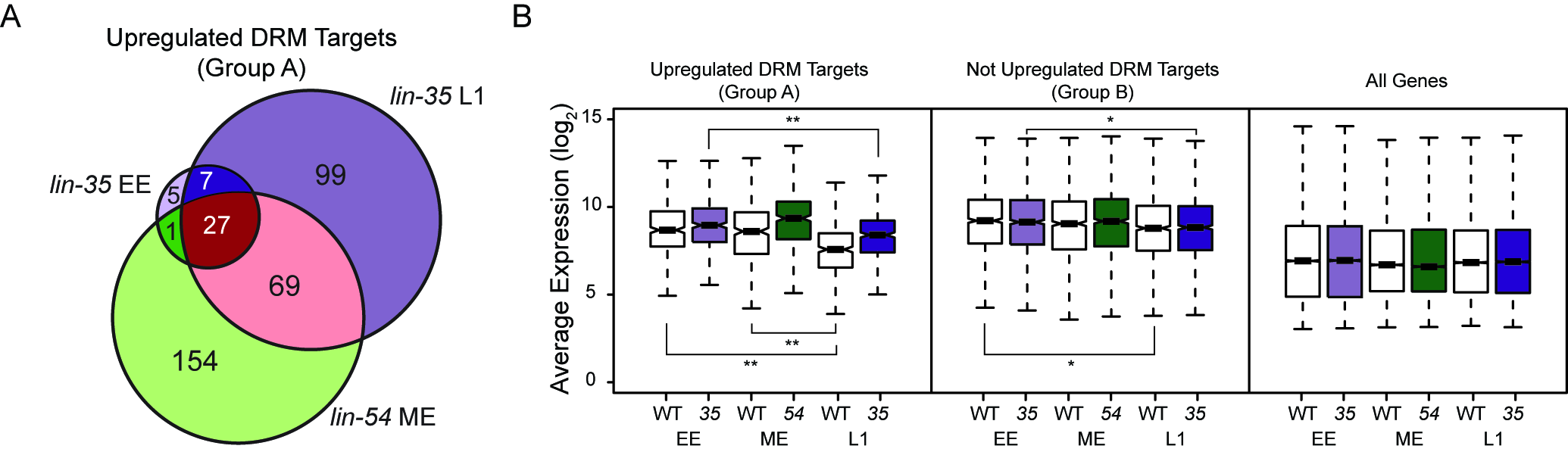

Supplement: S8 Fig — (A) Venn diagrams showing DRM target genes that are upregulated based on microarray analysis from [37] (lin-35 early embryo (EE) and L1, light and dark purple) and [33] (lin-54 mixed-stage embryo (ME), green) (Group A genes). (B) Box plots of wild-type (WT) or mutant (lin-35 or lin-54) log2 average expression values of Upregulated DRM targets (Group A), Not Upregulated DRM Targets (Group B), and All Genes. Staging of the samples for each experimental group is indicated: early embryos (EE), mixed-stage embryos (ME), and L1 larvae (L1). Box plots show the median (black bar) with each box extending from the 25th to 75th percentile. Extended whiskers indicate the 2.5th and 97.5th percentile, and wedges indicate the 95% confidence interval for the medians. Outliers were removed from the graphs. Significance (* p-value < 0.05, ** p-value < 0.01) was determined by a Wilcoxon-Mann-Whitney test. These results indicate that detectable upregulation reflects a drop in the average expression of DRM target genes in WT over developmental time, not an increase in the average expression of DRM target genes in mutants. This effect is also observed in Group B genes but the repression is not as dramatic. We speculate that Group A genes are progressively more repressed by DRM over developmental time; DRM dysfunction manifests as a failure in this repression and hence gene upregulation as development progresses. In contrast, Group B genes may include genes that 1) DRM does not fully repress or 2) are activated intermittently as development progresses. These findings suggest that detection of DRM target gene upregulation is dependent on developmental stage because wild-type DRM requires developmental time to establish repression of gene targets. (TIF) [file pgen.1007088.s008.tif]

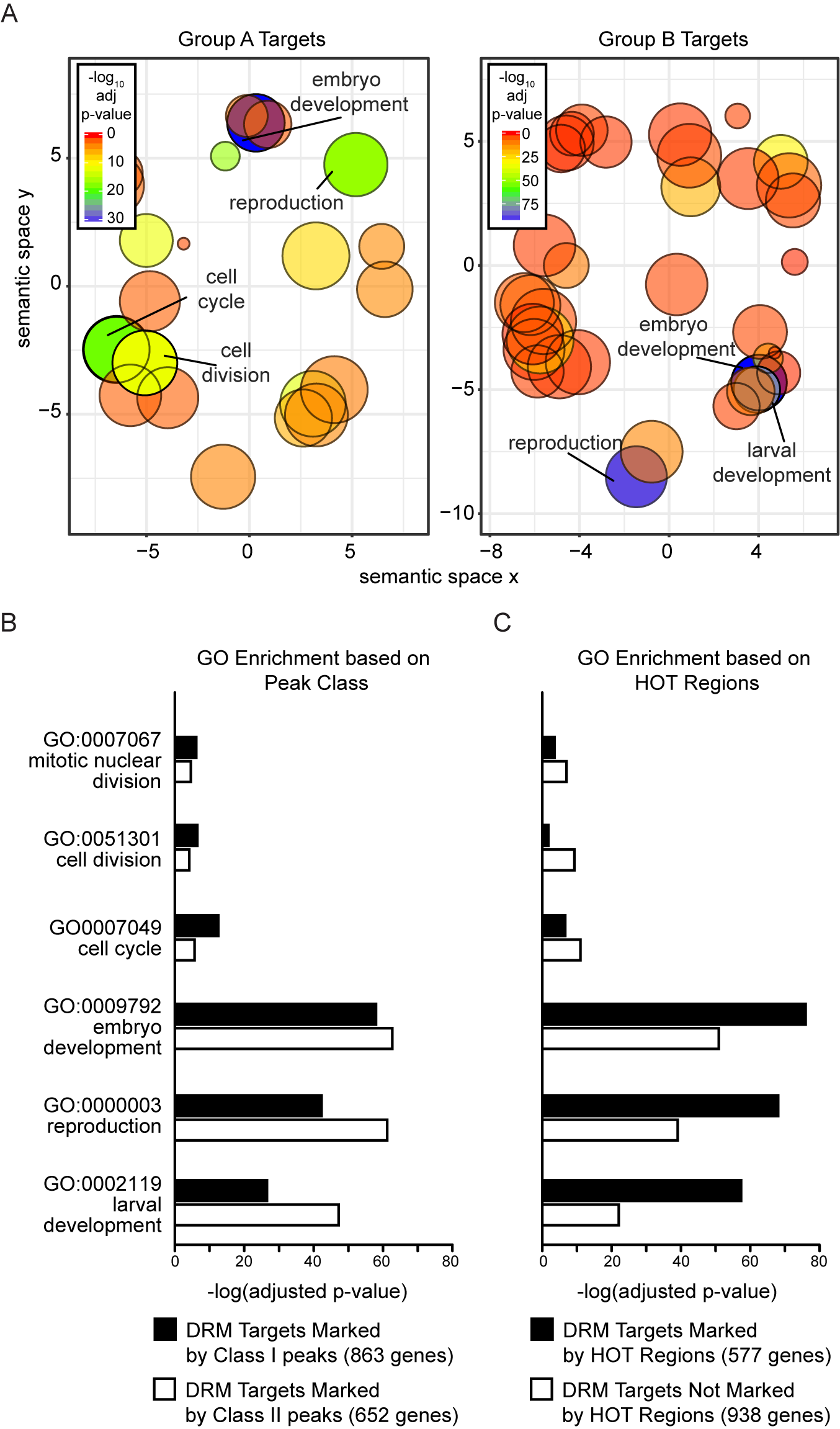

Supplement: S9 Fig — (A) REViGO gene ontology (GO) visualization of terms, which helped identify informative GO terms, enriched in Group A targets (left) vs. Group B targets (right), as described in Fig 5. GO terms are clustered on the x-y plane based on semantic similarity measures, with the size of each bubble indicating the GO term frequency and the color indicating the Benjamini adjusted p-value. Representative GO terms are highlighted and labeled. (B,C) Bar chart plotting the –log10 Benjamini adjusted p-value of selected GO term enrichment observed in (B) DRM targets marked by Class I peaks vs. Class II peaks, and (C) DRM targets marked by HOT regions vs. not HOT regions. (TIF) [file pgen.1007088.s009.tif]

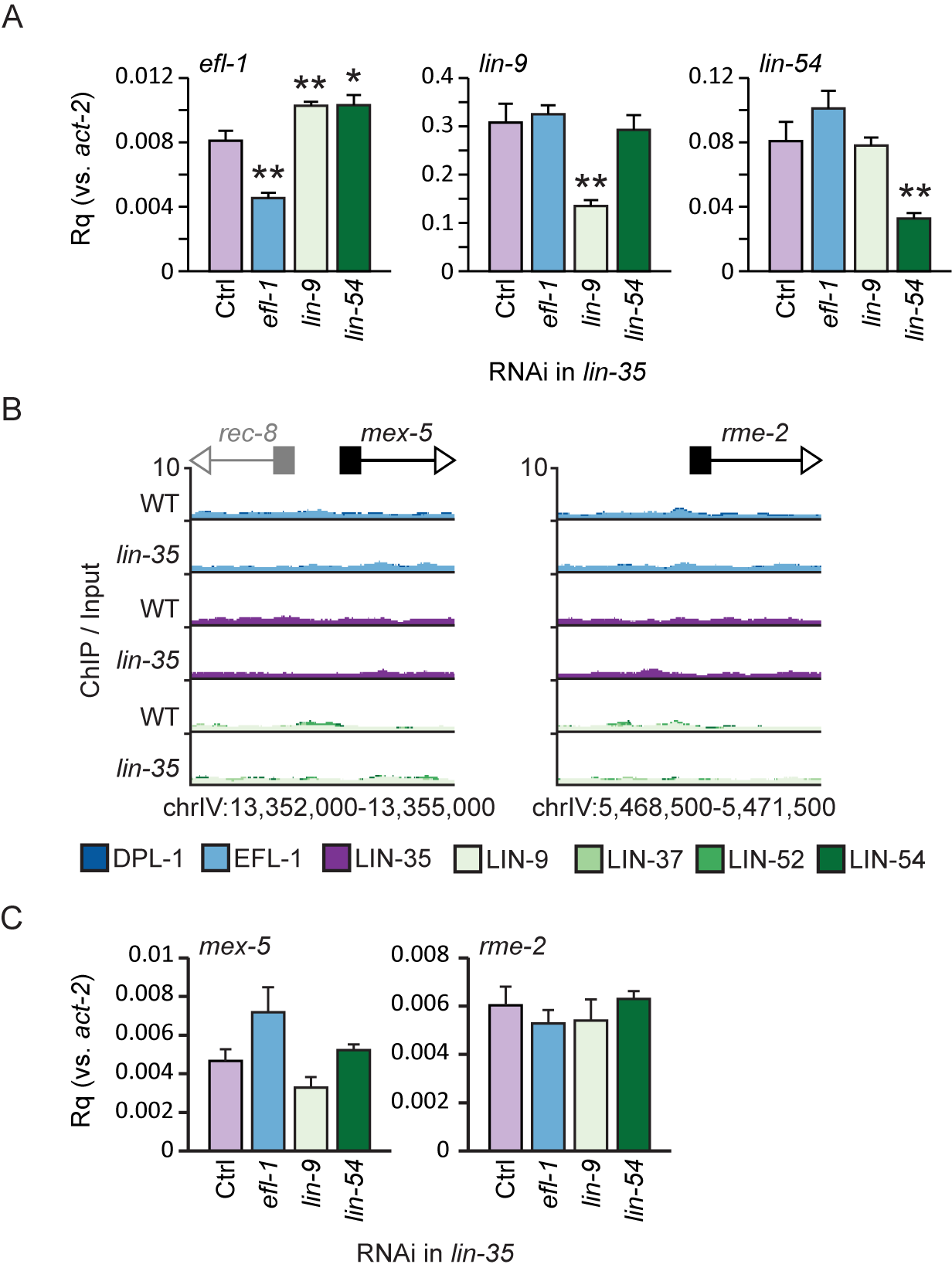

Supplement: S10 Fig — (A) RT-qPCR quantification of the effectiveness of RNAi depletion of efl-1 (blue), lin-9 (light green), and lin-54 (dark green) transcript levels in lin-35 null (lin-35(n745)) late embryos compared to empty-vector (Ctrl, purple) RNAi. (B) Genomic profiles of each DRM component in wild-type (WT) and lin-35 null late embryos at rme-2 and mex-5. E2F/DP subunits (blues), LIN-35 (purple) and MuvB subunits (greens) were overlaid on the same track. Normalized ChIP-seq enrichment values for each data track are indicated on the y-axis, and the chromosomal coordinates of the areas shown are indicated below the x-axis. Gene transcriptional start sites are indicated by black boxes, and coding strand directions are indicated by white arrowheads. (C) RT-qPCR analysis of rme-2 and mex-5 following efl-1 (blue), lin-9 (light green), and lin-54 (dark green) RNAi in lin-35 null late embryos as compared to empty-vector (Ctrl, purple). Expression values from 2 independent experiments each consisting of 4 biological replicates for each RNAi condition were averaged and presented as the relative quantity (Rq) compared to act-2. Error bars indicate standard error of the mean. (TIF) [file pgen.1007088.s010.tif]

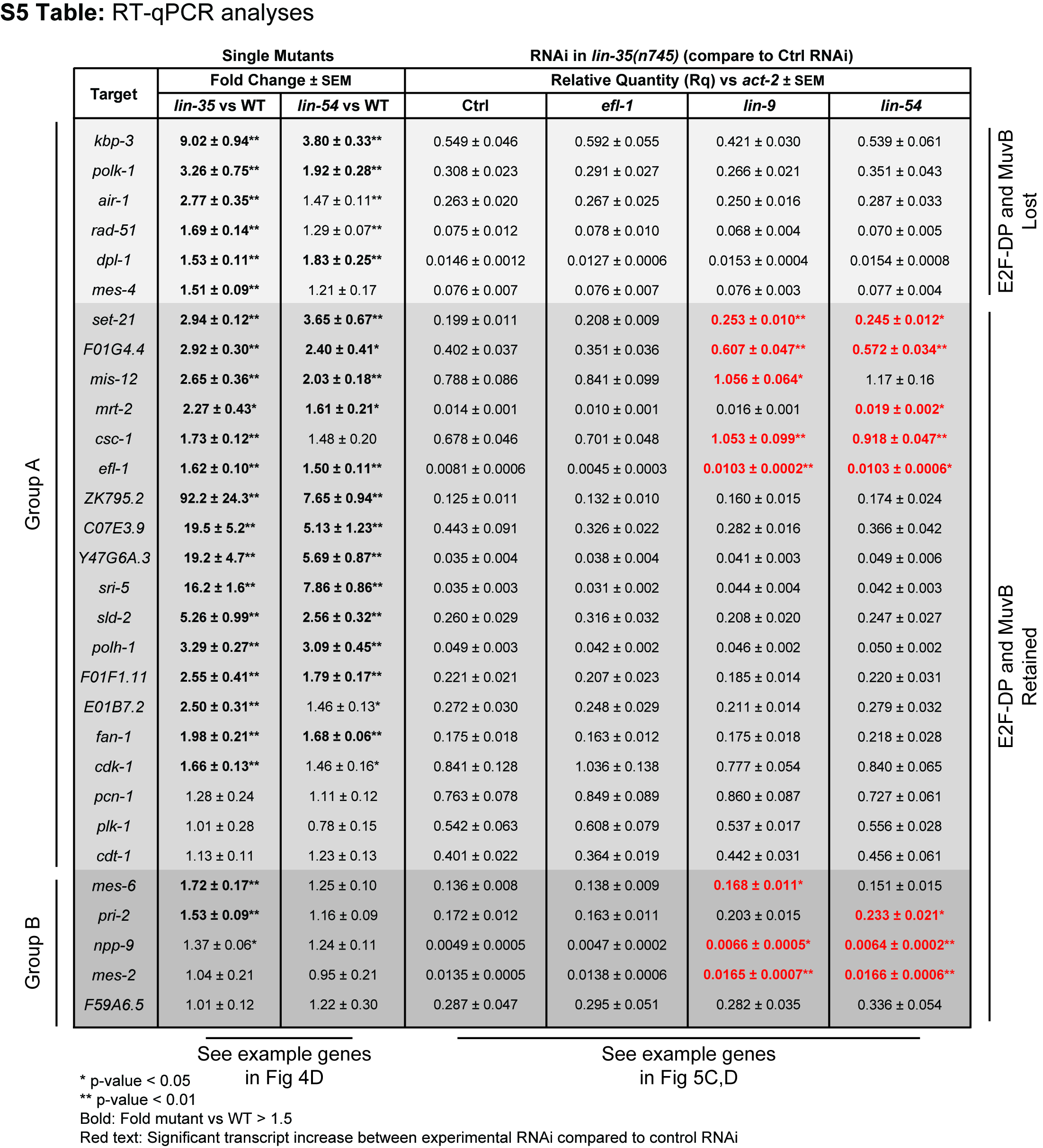

Supplement: S5 Table — (TIF) [file pgen.1007088.s015.tif]

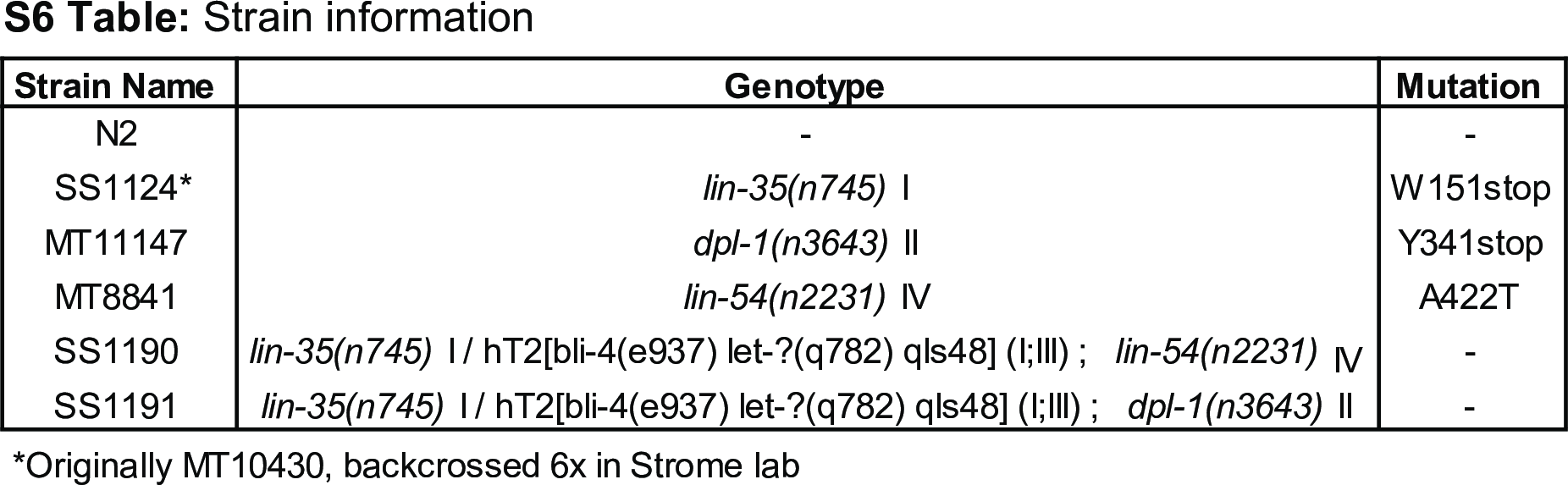

Supplement: S6 Table — (TIF) [file pgen.1007088.s016.tif]

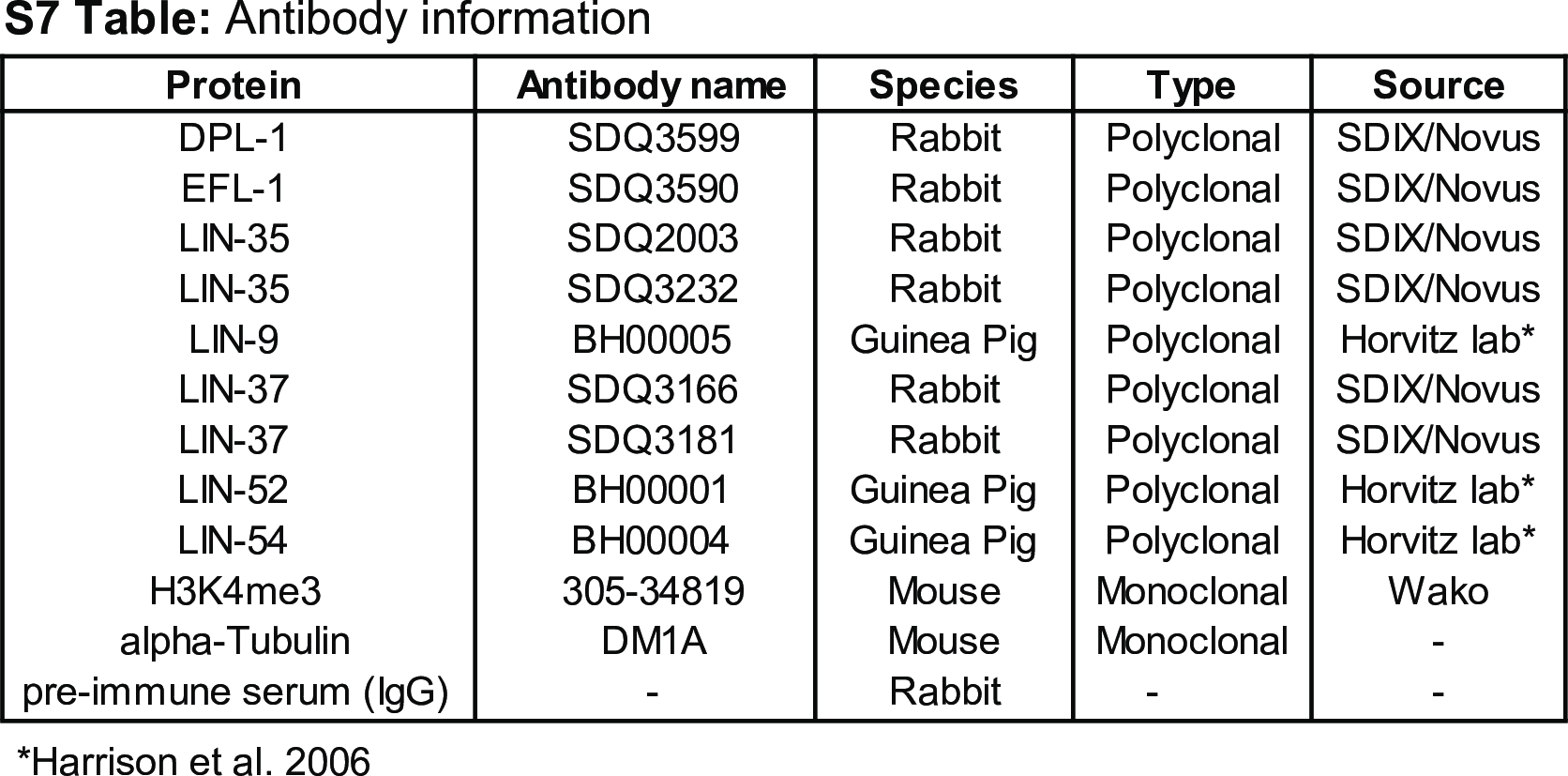

Supplement: S7 Table — (TIF) [file pgen.1007088.s017.tif]

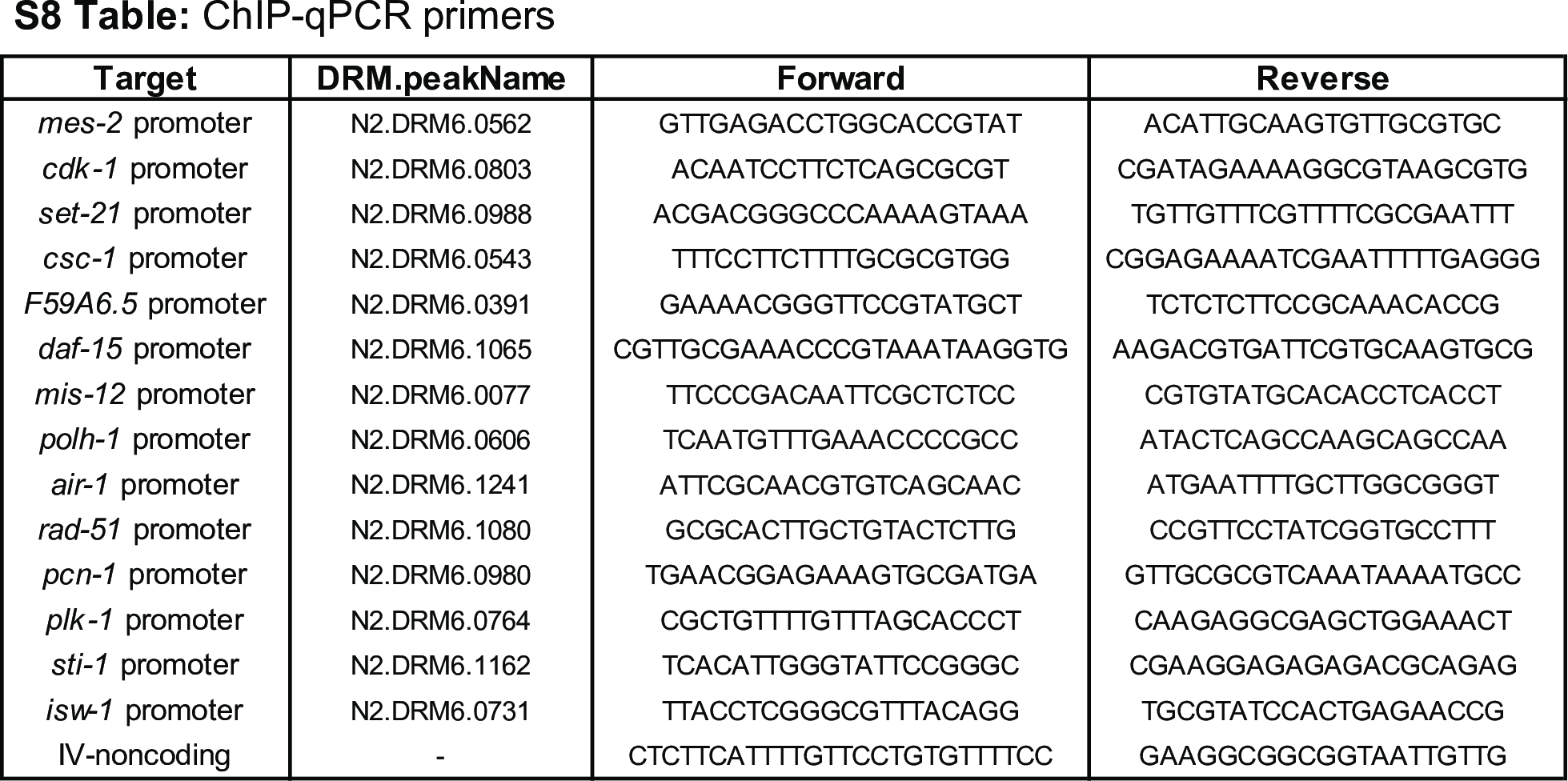

Supplement: S8 Table — (TIF) [file pgen.1007088.s018.tif]

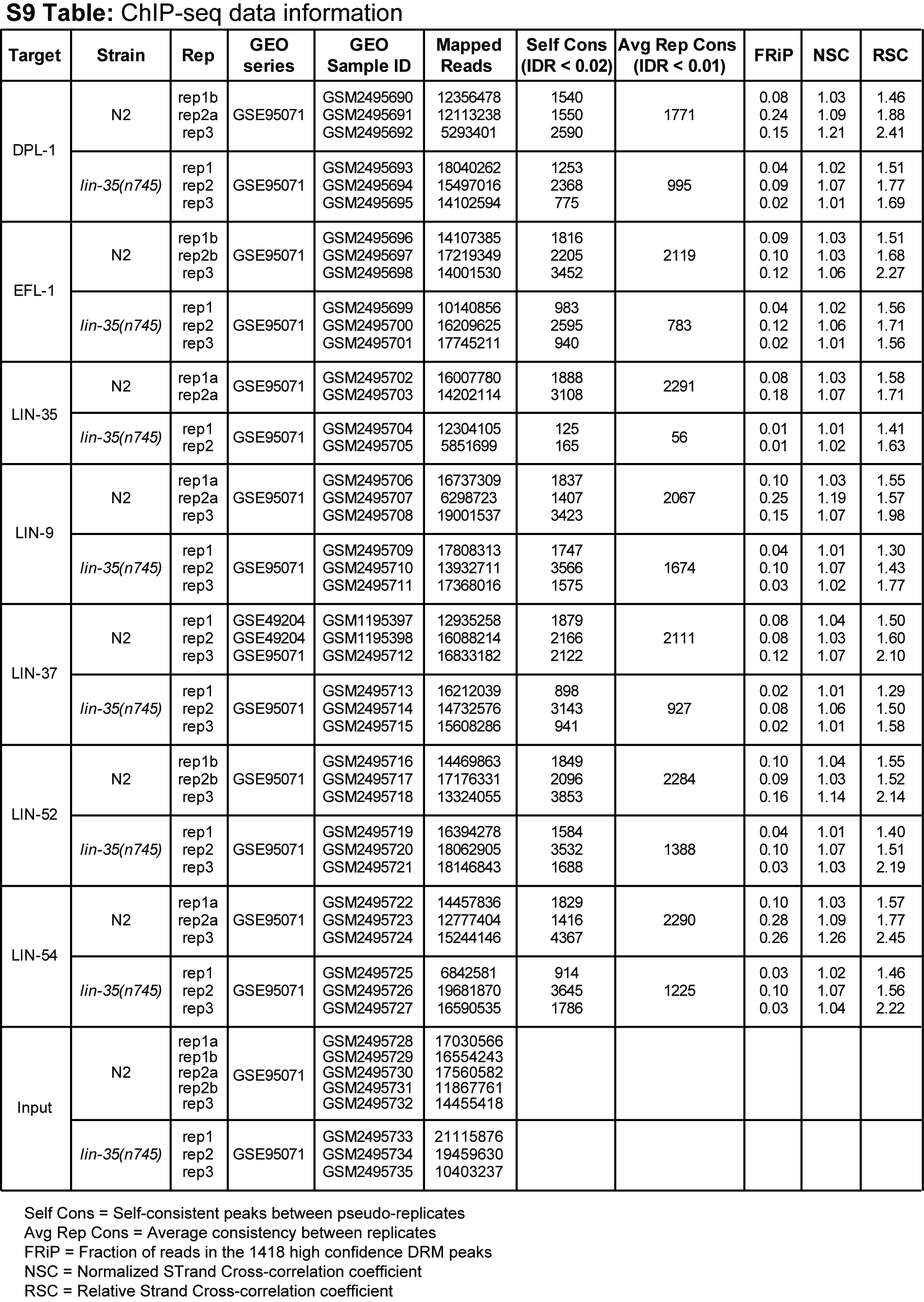

Supplement: S9 Table — (TIF) [file pgen.1007088.s019.tif]

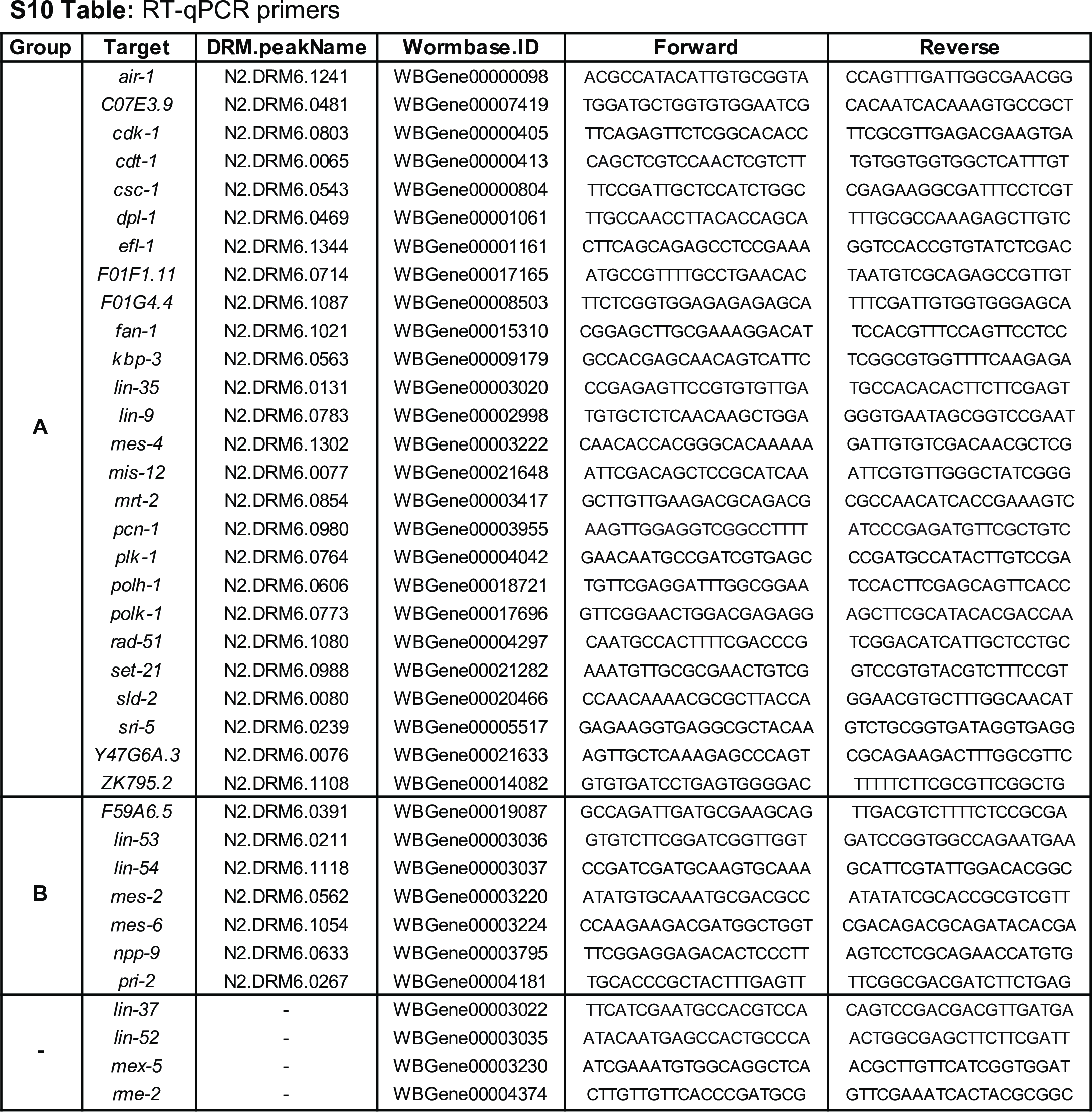

Supplement: S10 Table — (TIF) [file pgen.1007088.s020.tif]
